# Coagulative Granular Hydrogels with an Enzyme Catalyzed Fibrin Network for Endogenous Tissue Regeneration

**DOI:** 10.1002/adhm.202404146

**Published:** 2025-12-21

**Authors:** Zhipeng Deng, Camila B. Tovani, Simona Bianco, Gianni Comandini, Aya Elghajiji, Mina Aleemardani, Roxana Moscalu, Jeremie Zappia, Bianca Fernandes, Olivia Annett, Chrissy L. Hammond, Jason Wong, Dave J. Adams, Fabrizio Scarpa, Michael R. Whitehouse, Annela M. Seddon, James P. K. Armstrong

**Affiliations:** ^1^ Department of Translational Health Sciences, Bristol Medical School University of Bristol Bristol UK; ^2^ Laboratoire Chimie De La Matière Condensée De Paris Sorbonne Université Collège de France Paris France; ^3^ School of Chemistry University of Glasgow Glasgow UK; ^4^ Bristol Composites Institute School of Civil Aerospace and Design Engineering (CADE) University of Bristol Bristol UK; ^5^ Musculoskeletal Research Unit University of Bristol Bristol UK; ^6^ National Institute for Health Research Bristol Biomedical Research Centre University Hospitals Bristol and Weston NHS Foundation Trust and University of Bristol Bristol UK; ^7^ School of Physics HH Wills Physics Laboratory University of Bristol Bristol UK; ^8^ Blond McIndoe Laboratories, Division of Cell Matrix Biology and Regenerative Medicine, Faculty of Biology, Medicine and Health, School of Biological Sciences Manchester Academic Health Science Centre University of Manchester Manchester UK; ^9^ School of Physiology Pharmacology and Neuroscience University of Bristol Bristol UK

**Keywords:** double network hydrogel, fibrin, granular hydrogels, in situ gelation, injectable, microgels, thrombin

## Abstract

Granular hydrogels, composed of densely packed microgels, are an emerging class of injectable microporous scaffolds that provide interstitial porosity for endogenous cell recruitment and tissue repair. However, weak bonding interactions between constituent microgels compromise the mechanical integrity of these biomaterials, limiting their scope and effectiveness for in vivo applications where structural support is required. To address this challenge, we introduce a new bioinspired stabilization method and a novel class of regenerative biomaterial: *coagulative granular hydrogels*, assembled from thrombin‐functionalized gelatin methacryloyl microgels. The surface‐bound thrombin is enzymatically active and catalyzes the conversion of fibrinogen into a fibrin hydrogel that extends throughout the interstitial voids of the granular hydrogel. This secondary network acts as a biological glue to stabilize the granular hydrogel, yielding shear and compressive properties comparable to bulk hydrogel controls. Furthermore, the interstitial fibrin network provides a favorable microenvironment for the adhesion, proliferation, and invasion of endothelial cells, highlighting the potential of the biomaterial to support endogenous tissue repair. Subcutaneous injection in mice showed that the coagulative granular hydrogels preserved structural integrity and supported fibrin deposition, cell infiltration, and collagen remodeling in vivo. Future work will adapt this technology to other biomaterials and validate its performance for different tissue repair applications.

## Graphical abstract

*Coagulative granular hydrogels* are composed of packed thrombin‐functionalized microgels that catalyze the conversion of fibrinogen into a secondary fibrin network, filling the interstitial voids. This bio‐inspired approach stabilizes the biomaterial to match the robustness of bulk hydrogels without compromising injectability, mimicking the initial stage of wound healing and providing a conducive microenvironment for cell adhesion and proliferation.

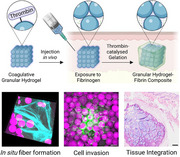

## 1 Introduction

Although the human body has a capacity for self‐healing, tissue damage is not always fully or correctly resolved to allow normal function. This has led to the development of regenerative biomaterials, engineered with biophysical and biochemical cues that can stimulate, accelerate, or guide endogenous tissue repair [1]. Polymer hydrogels are an attractive class of regenerative biomaterials providing highly hydrated 3D networks that can mimic native extracellular matrices and provide supportive and inductive environments for tissue repair [2]. However, the use of bulk hydrogels with continuous crosslinked polymer networks can present a number of challenges. For instance, bulk hydrogels are not generally injectable, and while it is possible to design systems that undergo sol‐gel transitions in vivo [3], this approach greatly constrains the choice of biomaterial. In addition, the dense nanoscale structure in bulk hydrogel networks can substantially limit host cell infiltration and nutrient mass transport [4].

These limitations have been addressed by an emerging class of biomaterials known as granular hydrogels, which consist of densely packed (jammed) microgels. These injectable biomaterials retain advantageous properties of bulk hydrogels (e.g., hydration, biochemical cues, nanoscale topography), while also providing an interstitial microporous network to allow the infiltration of endogenous cells and nutrients [5, 6]. These properties have driven extensive exploration of granular hydrogels as injectable porous biomaterials for regenerative medicine and tissue repair [7]. However, a major limitation of granular hydrogels is their substantially inferior compressive moduli and low shear yield stresses compared to bulk hydrogels of the same composition [8]. While a recent example using silk fibroin granular hydrogels achieved a shear storage modulus (G') of ≈ 70 kPa [9], this was the exception rather than the rule, with other non‐annealed granular hydrogels readily broken down by low‐level mechanical loading (G' ≈ 0.1–1 kPa) [10, 11, 12, 13, 14, 15, 16] and passively degrading under static incubation [17]. This represents an important limitation when applying granular hydrogels as in vivo scaffolds for endogenous tissue repair, a scenario that requires stability against disruption caused by physiological loads [18].

Many groups have sought to stabilize granular hydrogels by strengthening the interparticle interactions, generally by one of three different strategies. The first approach is to surface functionalize the component microgels with moieties that can undergo reversible binding, such as hydrazone bonding, electrostatic interactions, and host‐guest chemistries [12, 19, 20, 21]. This approach preserves injectability while stabilizing the static state; however, it provides only limited stabilization against shear forces (G' ≈ 1–5 kPa) [12, 17, 19, 20, 21]. The second approach is to form covalent bridges between the component microgels either via photocrosslinking or through the addition of enzymes, such as the use of factor XIII to anneal peptide‐functionalized granular hydrogels [22, 23, 24, 25]. This can provide greater shear stabilization (G' ≈ 1–10 kPa) [15, 26, 27, 28]; however, the need for external light sources or exogenous crosslinkers increases procedural complexity and limits translation as a minimally invasive injectable biomaterial. The third strategy is to fill the interstitial voids of the granular hydrogel with a secondary hydrogel network, an approach that has been used to improve shear stability [29], elastic modulus [30, 31, 32, 33], cyclic compression and stretch endurance [34]. However, the current approaches to incorporate a secondary network have either compromised the injectability of the granular hydrogel or required post‐curing; both practical factors that complicate clinical translation.

**FIGURE 1:**
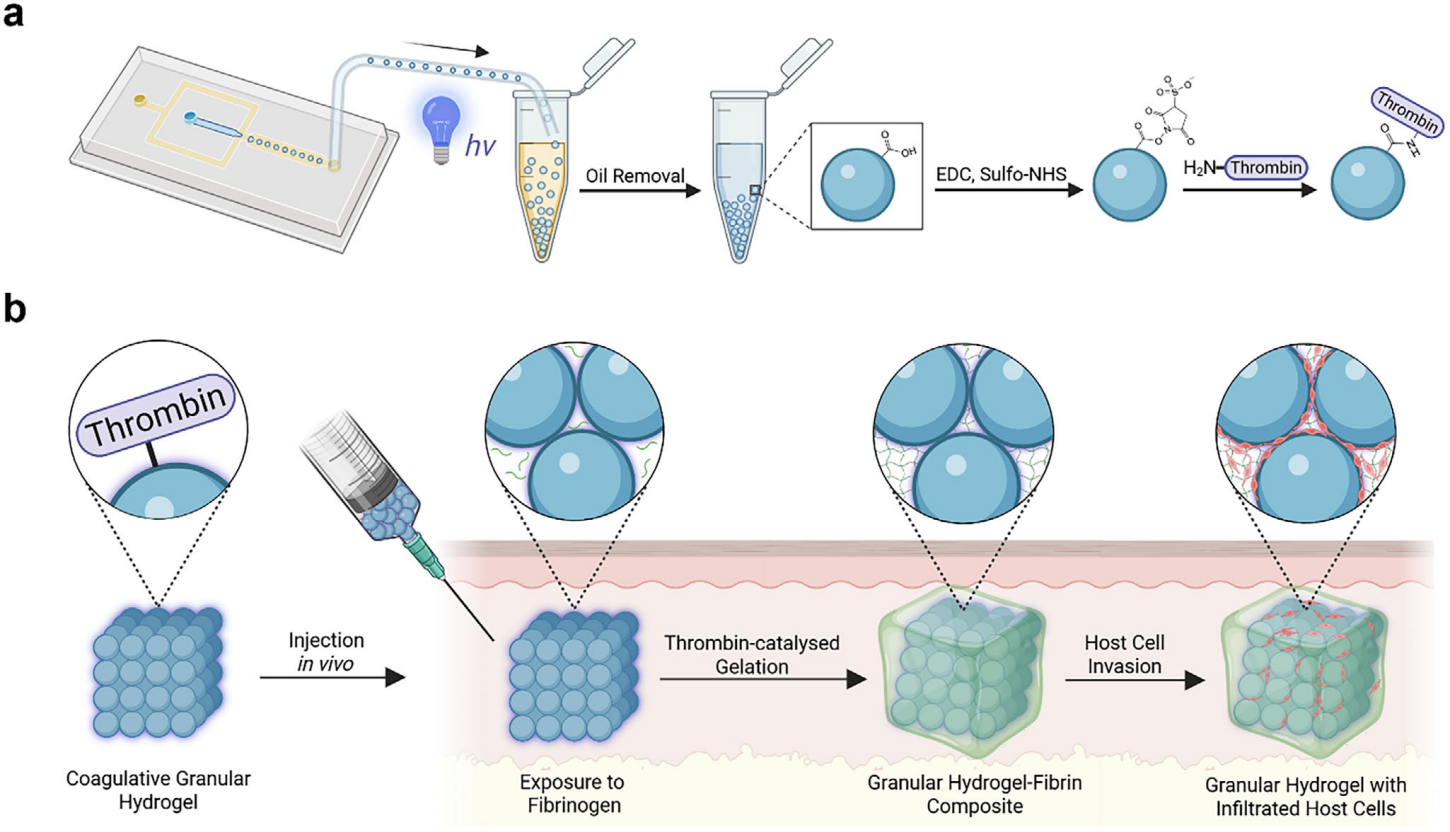
The fabrication and testing of coagulative granular hydrogel scaffolds. a) GelMA microgels are fabricated using emulsion microfluidics, transferred into an aqueous phase, and functionalized with thrombin using a 1‐ethyl‐3‐(3‐dimethylaminopropyl)carbodiimide (EDC) / *N*‐hydroxysulfosuccinimide (sulfo‐NHS) reaction. b) Thrombin‐functionalized microgels are assembled into a coagulative granular hydrogel, which can be administered via clinical‐grade needles. Exposure to fibrinogen molecules present in blood plasma results in the thrombin‐catalyzed formation of a fibrin secondary network that stabilizes the granular hydrogel and supports subsequent host cell infiltration.

Taking into board these considerations, we postulated whether a granular hydrogel system could be designed to evolve a secondary network after in vivo administration, specifically triggered by endogenous factors present within the body. This approach would allow a single‐component biomaterial with preserved injectability that can confer the stabilization needed for endogenous tissue repair without the need for any additional exogenous crosslinkers. To this end, we introduce a new bio‐inspired stabilization method and a novel class of regenerative biomaterial, namely *coagulative granular hydrogels* composed of thrombin‐functionalized microgels (Figure 1a). Thrombin is an important enzyme in wound healing that catalyzes the assembly of soluble blood plasma fibrinogen into cross‐linked fibrin networks [35]. The coagulative granular hydrogels harness this enzymatic reaction to catalyze the conversion of fibrinogen into a secondary fibrin network that fills the interstitial voids (Figure 1b). We have designed a robust fabrication method to produce coagulative GelMA granular hydrogels and have comprehensively assessed their enzymatic activity, composite structure, shear/compressive mechanics, and interactions with endothelial cells and spheroids. We have demonstrated that the fibrin network stabilizes the granular hydrogel, with the resulting composite providing compressive/shear mechanics on par with the bulk GelMA hydrogel of the same composition. Moreover, this fibrin hydrogel mimics the initial stage of wound healing, providing the granular hydrogel with a conducive microenvironment for cell adhesion and proliferation. We present a series of in vitro and in vivo studies that validate the biological response to the biomaterial.

## 2 Results and Discussion

### 2.1 Fabrication and Characterization of Thrombin‐Functionalized Microgels

**FIGURE 2:**
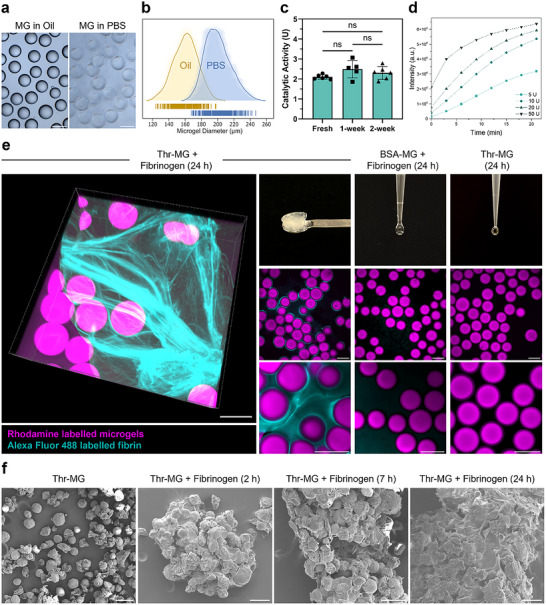
Characterization of thrombin‐functionalized GelMA microgels (Thr‐MG). a) Representative brightfield and phase contrast images of microgels in mineral oil and PBS, respectively. Scale bar: 200 µm. b) Average shifted histogram of the microgel diameter in mineral oil (160 ± 14 µm) and PBS (200 ± 16 µm), as measured from 317 microgels in both cases. c) Enzymatic activity of Thr‐MGs when freshly prepared (n = 6) and after storage at 4°C for 1 week (n = 5) and 2 weeks (n = 6). d) Enzymatic activity of Thr‐MGs with increasing thrombin used in the reaction (5, 10, 20, 50 U). e) Confocal fluorescence microscopy and camera images of Thr‐MG after 24 h of fibrinogen incubation, as well as controls of BSA‐MG with fibrinogen and Thr‐MG without fibrinogen. The microscopy study was performed using rhodamine‐functionalized microgels (magenta) and Alexa Fluor 488 conjugated fibrinogen (cyan). Scale bar: 200 µm. f) SEM images of Thr‐MGs alone, and after 2, 7, and 24 h of incubation with fibrinogen. Scale bar: 100 µm.

We used an established protocol to synthesize GelMA using gelatin and methacrylic anhydride [36]. Proton nuclear magnetic resonance (^1^H NMR) spectroscopy was used to confirm the substitution of the free gelatin amines (Figure S1) [37], while a fluoraldehyde assay confirmed a consistent degree of functionalization of 91 ± 2% across the five batches used in this study (Figure S2). We designed and made a microfluidic chip with a flow‐focusing geometry (Figure S3), which we used to generate size‐tunable microdroplets containing an aqueous phase of 8% w/v GelMA solution surrounded by a continuous phase of surfactant‐stabilized mineral oil. The inclusion of 8.5 mm lithium phenyl (2,4,6‐trimethylbenzoyl) phosphinate (LAP) in the aqueous polymer phase enabled efficient photocrosslinking of the microdroplets into GelMA microgels, which were washed and then phase transferred into phosphate‐buffered saline (PBS). The crosslinked GelMA microgels were spherical, with a diameter of 160 ± 14 µm in oil, which increased to 200 ± 16 µm in PBS due to the swelling of polymer chains in aqueous solution (Figures 2a,b).

We used carbodiimide‐based chemistry to immobilize thrombin on the surface of the GelMA microgels. The activity of the surface‐immobilized thrombin was evaluated using a 7‐amino‐4‐methylcoumarin fluorometric assay (Figure 2c). Enzymatic activity was detected in the thrombin‐functionalized microgels (Thr‐MG) but not in the surrounding buffer, indicating a successful coupling reaction (Figure S4a). When 5 U of thrombin was used, 42% of the catalytic activity was preserved after crosslinking, with minimal variation across six independent batches (± 0.2%). This value was similar to controls in which thrombin was incubated overnight without any microgels or crosslinking reagents (43%), suggesting that the observed reduction in activity was due to the overnight incubation rather than conjugation to the microgels (Figure S4b). The surface‐bound thrombin remained stable at 4°C, with no significant loss of activity observed after two weeks of incubation. This thrombin concentration, which was used for all subsequent experiments, yielded Thr‐MGs with an enzymatic activity of ∼0.12 U mg^−1^ (by dry mass). However, it should also be noted that the enzymatic activity of Thr‐MGs could be increased by raising the concentration of thrombin during the functionalization reaction (Figure 2d), which could, in theory, allow faster catalysis for different biomedical applications (e.g., as a hemostatic biomaterial). Importantly, there was negligible enzymatic activity in both control groups: unfunctionalized microgels (MG) and microgels functionalized with bovine serum albumin (BSA‐MG).

We next sought to investigate the interaction between Thr‐MG and the fibrinogen substrate at 37°C. Brightfield microscopy showed that fibrinogen induced the aggregation of Thr‐MG after just 15 min (Figure S5) to form a stable gel after 24 h (Figure 2d). High magnification brightfield images revealed fibers surrounding the Thr‐MGs (Figure S6), which suggested that the soluble fibrinogen had been successfully catalyzed into a secondary fibrin network that effectively “glued” the microgels together. Interestingly, microgel controls without thrombin also formed loose aggregates following the addition of fibrinogen, but did not form a gel after 24 h. These results indicated that fibrinogen molecules induced the early aggregation of microgels, while the catalysis of fibrinogen into fibrin was mediated by the surface‐immobilized thrombin. We next used confocal fluorescence microscopy to visualize fluorescently labelled Thr‐MGs exposed for 24 h to a fluorescently‐conjugated fibrinogen. These images showed bright halos of fibrin around the Thr‐MGs, while 3D rendering clearly revealed a dense fibrillar network surrounding and bridging the microgels (Figure 2d and Video S1). This network was present throughout the full volume of the Thr‐MG sample but was absent in the control groups (BSA‐MGs with fibrinogen, Thr‐MGs without fibrinogen).

Scanning electron microscopy (SEM) was used to examine the development of the fibrin gel at different time points after the addition of fibrinogen (2, 7, 24 h) (Figure 2e). The Thr‐MGs were initially discrete microgels with smooth surfaces and open pores, but 2 h of incubation with fibrinogen produced small aggregates covered in fibers. After 7 and 24 h, these fibers had assembled into larger strands and bundles, connecting the microgels to form a densely packed fibrillar network. High magnification images confirmed that the fibril morphology in the Thr‐MG samples closely matched a bulk fibrin gel control (Figure S7). In contrast, no fibers were observed in the control groups without thrombin functionalization (Figure S8). Taken together, the confocal fluorescence microscopy and SEM images provided strong evidence that the formation of fibrin fibers was specifically catalyzed by the surface‐immobilized thrombin.

### 2.2 Coagulative Granular Hydrogel Scaffold

**FIGURE 3:**
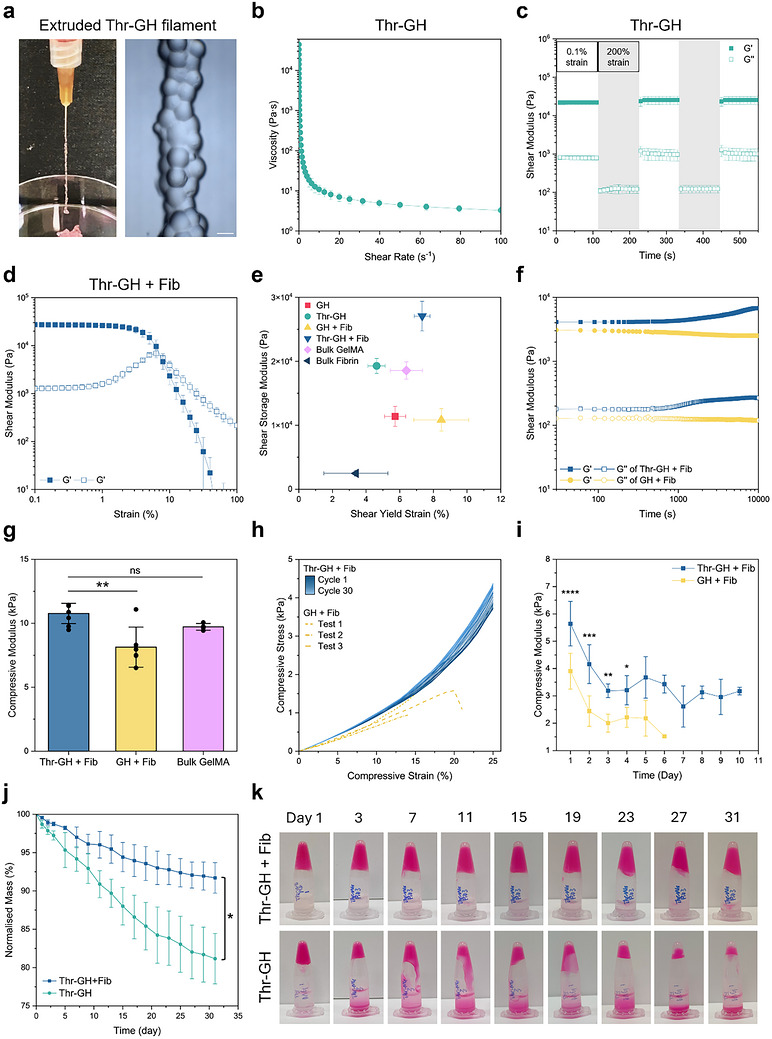
Coagulative granular hydrogel rheology, compression testing, and stability assays. a) Camera image and representative brightfield image of a thrombin‐functionalized granular hydrogel (Thr‐GH) manually extruded through a 26‐gauge needle. Scale bar: 200 µm. b) Viscosity measurement of Thr‐GH with shear rates from 0–100 s^−1^. c) Shear recovery test of Thr‐GH, measuring shear storage modulus (G') and loss modulus (G'') at alternating 0.1% strain (unshaded) and 200% strain (shaded). d) Strain sweep of Thr‐GH‐fibrin composite (Thr‐GH + Fib), measuring G' and G'' across 0.1%–100% shear strain. e) Comparison of G' and yield strain of Thr‐GH + Fib and controls. f) Time‐resolved rheology showing the change in G' and G'' after addition of fibrinogen to GH or Thr‐GH. g) Characterization of the dynamic compressive moduli of Thr‐GH + Fib, GH+ Fib, and a bulk GelMA hydrogel, calculated as the slope between 2–7% strain. Statistical analysis performed using the Kruskal‐Wallis test with Dunn's post‐hoc test. ^**^ *p* < 0.01, ns = no significance (p > 0.05). h) Cyclic compression tests of Thr‐GH + Fib under dynamic cyclic loading from 0–25% strain for 30 cycles. The stress–strain curves of GH + Fib samples failed to reach 25% strain on the first cycle (shown as a yellow dashed line). i) Changes in the compressive modulus of Thr‐GH + Fib (n = 5 throughout) and GH + Fib (n = 5 on day 1) samples incubated at 37°C for 10 days, with moduli assessed daily and calculated as the slope between 2–7% strain. GH + Fib samples began to progressively dissociate, leading to an increasing number of untestable samples on day 2–3 (n = 4), day 4 (n = 3), day 5 (n = 2), and day 6 (n = 1). Statistical analysis between the two groups was performed using a Two‐Way ANOVA (mixed‐effects model) with Tukey's post‐hoc test up to day 4. ^****^ *p* < 0.0001, ^***^ *p* < 0.001, ^**^ *p* < 0.01, ^*^ *p* < 0.05. j) Mass normalized to day 0 (%) of Thr‐GH + Fib and Thr‐GH over 31 days of incubation in PBS at 37°C. Statistical analysis performed using a Two‐Way ANOVA with Tukey's post‐hoc test, significance between the two groups on day 31 was shown. ^*^ *p* < 0.05. k) Camera images showed the dissociation of Thr‐GH from early timepoints, whereas Thr‐GH + Fib stayed as an intact structure over 31 days. For viscosity, shear recovery test, strain sweeps, storage moduli, yield strains, compressive moduli, and mass change, data are presented as mean ± standard deviation, with a sample size of n ≥ 3.

Having verified the enzymatic activity of the Thr‐MGs, we used centrifugation to form jammed granular hydrogels (Thr‐GH). The granular hydrogel could be readily extruded by hand through a 26‐gauge needle (inner diameter = 0.26 mm) to form long, continuous strands (Figure 3a). Brightfield microscopy images of the extruded Thr‐GH filaments confirmed that the individual microgels remained intact. These results are consistent with studies that use granular hydrogels as injectable biomaterials that flow at high strain and then rapidly recover into an elastic solid at low strain [10, 19, 38, 39]. Shear thinning was confirmed by rheology, which was used to measure a sharp decrease in the viscosity of Thr‐GH with increasing shear rate (Figure 3b). The samples were next subjected to cycles of low and high strain in order to assess shear recovery (Figure 3c). The Thr‐GH behaved like an elastic solid at 0.1% strain, with a storage modulus (G′ = 21.9 ± 2.9 kPa) substantially higher than the loss modulus (G′′ = 0.8 ± 0.1 kPa). The Thr‐GH rapidly transitioned to a liquid‐like state at 200% strain (G′ = 0 kPa, G′′ = 0.1 ± 0.0 kPa) and then quickly recovered when returned to 0.1% strain (first cycle G′ = 25.3 ± 6.3 kPa, G′′ = 1.0 ± 0.3 kPa; second cycle G′ = 25.3 ± 6.0 kPa, G′′ = 1.0 ± 0.4 kPa). This non‐Newtonian behavior, dependent on interparticle interaction and friction [6, 16, 39], matched the unfunctionalized granular hydrogel (GH) controls (Figure S9), which confirmed that the shear thinning and recovery were not affected by thrombin functionalization.

Rheology was also used to quantify the dynamic shear mechanical properties of the Thr‐GH‐fibrin composite (Thr‐GH + Fib) with granular and bulk hydrogel controls. The profile of the strain/frequency sweeps was consistent with the literature [16, 40, 41], presenting linear trends in G′ at low frequency as well as broad linear viscoelastic regions with strain‐independent G′ (Figure 3d, Figures S10 and S11). While the yield strain was similar for all samples (between 5 and 8%), the G′ of Thr‐GH + Fib (27.1 ± 2.3 kPa) was significantly higher than the five controls: Thr‐GH (19.3 ± 1.2 kPa), GH + Fib (10.8 ± 1.7 kPa), GH (11.4 ± 1.6 kPa), bulk GelMA hydrogels (18.6 ± 1.3 kPa), and bulk fibrin hydrogels (2.5 ± 0.4 kPa) (Figure 3e). These results indicated that the secondary fibrin network throughout Thr‐GH dissipates stress under shear, leading to an improved shear modulus that exceeded even the bulk hydrogel controls. We also used time‐resolved rheology to monitor the temporal changes in the granular hydrogels during incubation with fibrinogen (Figure 3f). Thr‐GH showed increases in G′ and G′′ after 500 s that eventually plateaued at ∼10 000 s, while the shear moduli values of the GH control remained stable throughout.

While these rheology studies confirmed the response to shear, it was important to understand how the biomaterial would respond to compressive loads that might be present in vivo [42]. Dynamic compression tests were performed under wet conditions to mimic physiological conditions [43]. The compressive modulus of Thr‐GH + Fib (10.8 ± 0.8 kPa) was significantly higher than the GH + Fib control (8.1 ± 1.6 kPa) and, notably, was comparable to the bulk GelMA hydrogel control (9.7 ± 0.3 kPa) (Figure 3g). We next sought to repeatedly compress the granular hydrogels to 25% strain to understand the biomaterial response to cyclic loading. The GH + Fib controls all disintegrated at the first loading cycle, with failure measured between 14–21% strain. In contrast, we successfully compressed the Thr‐GH + Fib composite to 25% strain over 30 cycles without failure (Figure 3h). The compressive moduli of Thr‐GH + Fib samples were also tested using granular hydrogels formed using different quantities of thrombin. A small increase was observed when thrombin was increased from 5 to 10 U (7.3 ± 0.3 kPa and 8.3 ± 1.0 kPa, respectively; Figure S12); however, the difference was not statistically significant. This was anticipated, given that increases in thrombin concentration are likely to affect the catalytic rate rather than the final fibrin concentration [35].

Static compression tests were used to monitor changes in the compressive modulus of Thr‐GH + Fib under daily dynamic loading over a 10‐day incubation period at 37°C (Figure 3i). On day 1, the compressive modulus of Thr‐GH + Fib (5.6 ± 0.7 kPa) was significantly higher than the GH + Fib control (4.0 ± 0.7 kPa). Although both groups exhibited a gradual decrease in compressive modulus over time, the Thr‐GH + Fib samples plateaued at 3.2 ± 0.2 kPa by day 3. In contrast, the GH + Fib samples began to lose shape fidelity from day 2 and progressively dissociated, with one final measurable compressive modulus recorded on day 6 (1.5 kPa). To isolate the effects of mechanical loading from passive degradation, both sample types were also incubated statically at 37°C for 10 days and tested only at the end of the period (Figures S13 and S14). Similar trends were observed, suggesting that changes in compressive modulus were primarily due to incubation rather than daily mechanical loading.

Stability against disaggregation was visually demonstrated by subjecting the granular hydrogels to continuous agitation and imaging the samples over time. While the Thr‐GH control disintegrated after 15 min of agitation and completely dissociated after 2 h, the Thr‐GH + Fib composite remained stable throughout (Figure S15). We next carried out a passive degradation experiment by repeatedly measuring the mass of the Thr‐GH + Fib and granular hydrogel controls (Thr‐GH and GH) over a one‐month period in the absence of any external agitation or loading (Figure 3j). The normalized mass ratio of Thr‐GH + Fib on day 31 compared to the original mass was 91 ± 2%, significantly higher than the corresponding value for the Thr‐GH control (81 ± 2%). It was worth noting that under visual inspection, the Thr‐GH + Fib remained intact throughout the testing period with only small quantities dissociating from the granular hydrogel over time (Figure 3k). In contrast, the Thr‐GH control started to dissociate from day 1 and day 3, respectively, with large quantities of microgels breaking from the granular hydrogel and flowing into the supernatant. Collectively, these results indicated that the Thr‐GH + Fib composite could endure repeated compression at high strain, with the secondary fibrin network significantly improving the compressive moduli and stabilizing the granular hydrogel under static and dynamic conditions.

**FIGURE 4:**
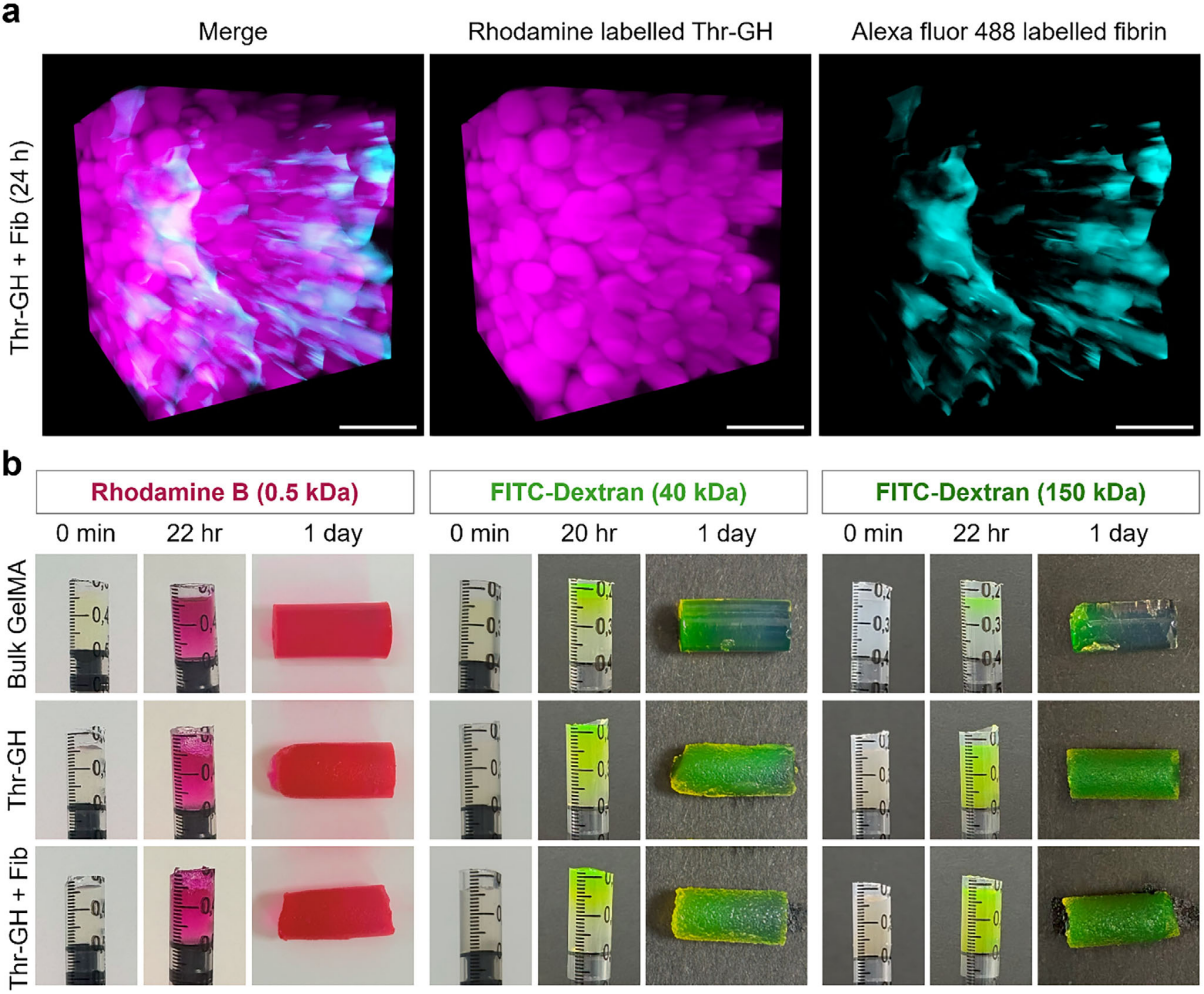
Volumetric imaging and molecular infiltration of coagulative granular hydrogels. a) 3D reconstructed stacks of Thr‐GH + Fib using light sheet fluorescence microscopy. Thr‐GHs were made using GelMA that was fluorescently labelled with rhodamine (shown in magenta) and incubated with Alexa Fluor 488 conjugated fibrinogen (shown in cyan). Scale bar: 500 µm. b) Camera images showing the infiltration of Rhodamine B (0.5 kDa) and FITC‐labelled dextran (40 kDa and 150 kDa) over time in 10‐mm deep Thr‐GH + Fib samples compared to bulk GelMA hydrogel and Thr‐GH controls.

The microgel packing density is a critical property of granular hydrogels that significantly impacts mechanical properties as well as the ability to support cell and vessel infiltration [44]. Volumetric reconstruction of light sheet fluorescence microscopy images (Figure 4a and Figure S16) was used to calculate a microgel packing density of 0.67 for Thr‐GH. This value suggested that the deformable nature of the microgels enabled a packing density midway between the theoretical maxima for hard spheres in random close‐packed (0.64) and cubic/hexagonal close‐packed (0.74) configurations [45]. Interestingly, the Thr‐GH + Fib composite had a lower microgel packing density (0.54), with the interstitial space filled by fibrin. This finding indicated that the formation of fibrin fibers pushes apart the microgels slightly, thus increasing the void fraction compared to Thr‐GH alone. This expansion provides a new approach to increase the void fraction without compromising the structural integrity and mechanical properties of the granular hydrogels.

One concern was that the presence of a secondary fibrin network might inhibit molecular diffusion and negate a key benefit of granular hydrogels. To assess molecular permeability, fluorescent molecules of varying molecular weights (0.5 kDa, 40 kDa, 150 kDa) were introduced to the surface of 1 mm thick Thr‐GH + Fib samples, with the infiltration throughout the biomaterial imaged after 1 d and compared with Thr‐GH and bulk GelMA controls (Figure 4b and Figures S17 and S19). Rhodamine B, with a molecular weight of ∼0.5 kDa, fully infiltrated all three biomaterials within 24 h. In contrast, the passage of the 40 and 150 kDa FITC‐labelled dextrans did appear to be more restricted in Thr‐GH + Fib compared to the Thr‐GH control; however, both granular hydrogels exhibited greater infiltration than the bulk GelMA control. While infiltration will be dependent on molecular size, charge, and fluid flow conditions, the tested conditions suggest that the fibrin network may enable small molecule passage (0.5 kDa) while partially hindering larger macromolecule diffusion (40–150 kDa).

### 2.3 In Vitro Cell Culture

**FIGURE 5:**
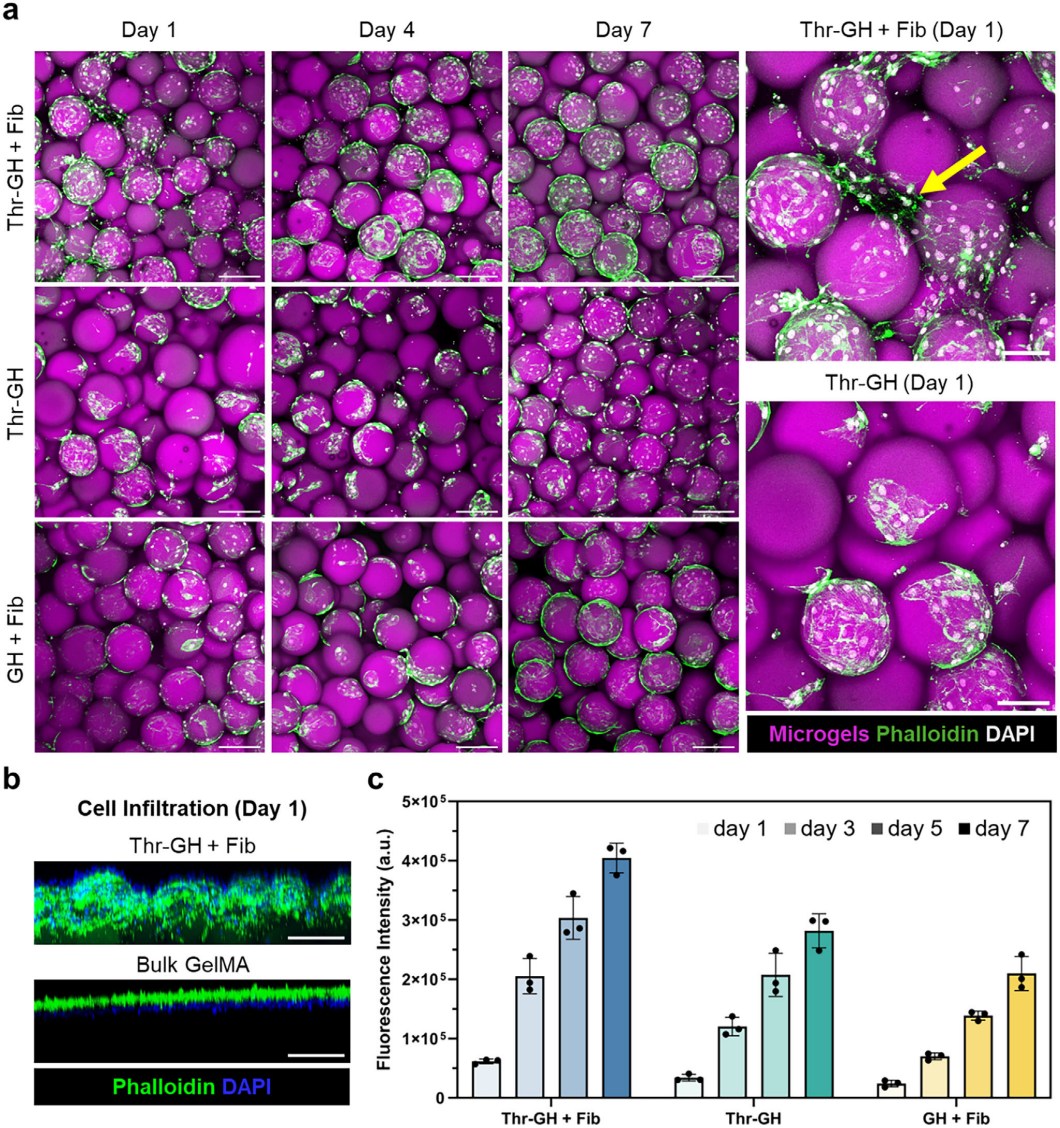
In vitro cell culture. a) Representative maximum intensity projection images of HUVECs cultured on Thr‐GH + Fib, Thr‐GH, and GH + Fib over 7 days. HUVECs exhibited spreading in the interstitial fibrin network of Thr‐GH + Fib (indicated with a yellow arrow), which was not seen in the Thr‐GH control. Granular hydrogels were made using GelMA that was fluorescently labelled with rhodamine (shown in magenta), while the cells were stained for F‐actin filaments (phalloidin, shown in green) and nuclei (DAPI, shown in white). Scale bar: 200 µm. b) Representative maximum intensity projection images resliced from confocal fluorescence microscopy stacks revealing the infiltration of HUVECs into Thr‐GH + Fib compared to the bulk GelMA hydrogel control. The cells were stained for F‐actin filaments (phalloidin, green) and nuclei (DAPI, blue). Scale bar: 200 µm. c) Quantification of cell metabolism in Thr‐GH + Fib, Thr‐GH, and GH + Fib over 7 days. All data are presented as mean ± standard deviation, with a sample size of n = 3.

The interstitial fibrin network was designed not only to stabilize the granular hydrogel but also to support endogenous cell regeneration. We thus performed a series of in vitro cell studies to model whether the Thr‐GH + Fib composite would support the adhesion, infiltration, and proliferation of endothelial cells. We seeded human umbilical vein endothelial cells (HUVECs) on the surface of Thr‐GH + Fib and compared the cell response to granular hydrogel controls (Thr‐GH, GH + Fib). Samples were fixed on days 1, 4, and 7 of culture, stained for F‐actin and DNA, imaged with multiphoton microscopy, then used to capture a z‐stack of the cells at the top surface of the granular hydrogel (Figure 5a and Figure S20). This qualitatively showed more cells present on the Thr‐GH + Fib samples than the controls after 1 day of culture, which suggests that the fibrin network aided cell adhesion to the biomaterial. All samples supported cell proliferation, with an increasing number of cells and coverage of the microgels over a week; however, there were notable differences in the location and morphology of the cells. In particular, the Thr‐GH + Fib samples exhibited endothelial cells spreading throughout the interstitial fibrin network, whereas the cells on the granular hydrogel controls were bound exclusively to the microgel surface. Resliced confocal microscopy images revealed that HUVECs had infiltrated into the cross‐sectional area of the Thr‐GH + Fib, whereas in bulk GelMA of the same concentration, the cells remained attached to the surface and did not invade into the hydrogel (Figure 5b). AlamarBlue assays performed on days 1, 3, 5, and 7 showed an increase in total metabolic activity for all samples, which provided an indirect confirmation of cell proliferation (Figure 5c and Figure S21) [46, 47]. Collectively, these in vitro results demonstrate that Thr‐GH + Fib is capable of supporting the adhesion, infiltration, and proliferation of endothelial cells.

**FIGURE 6:**
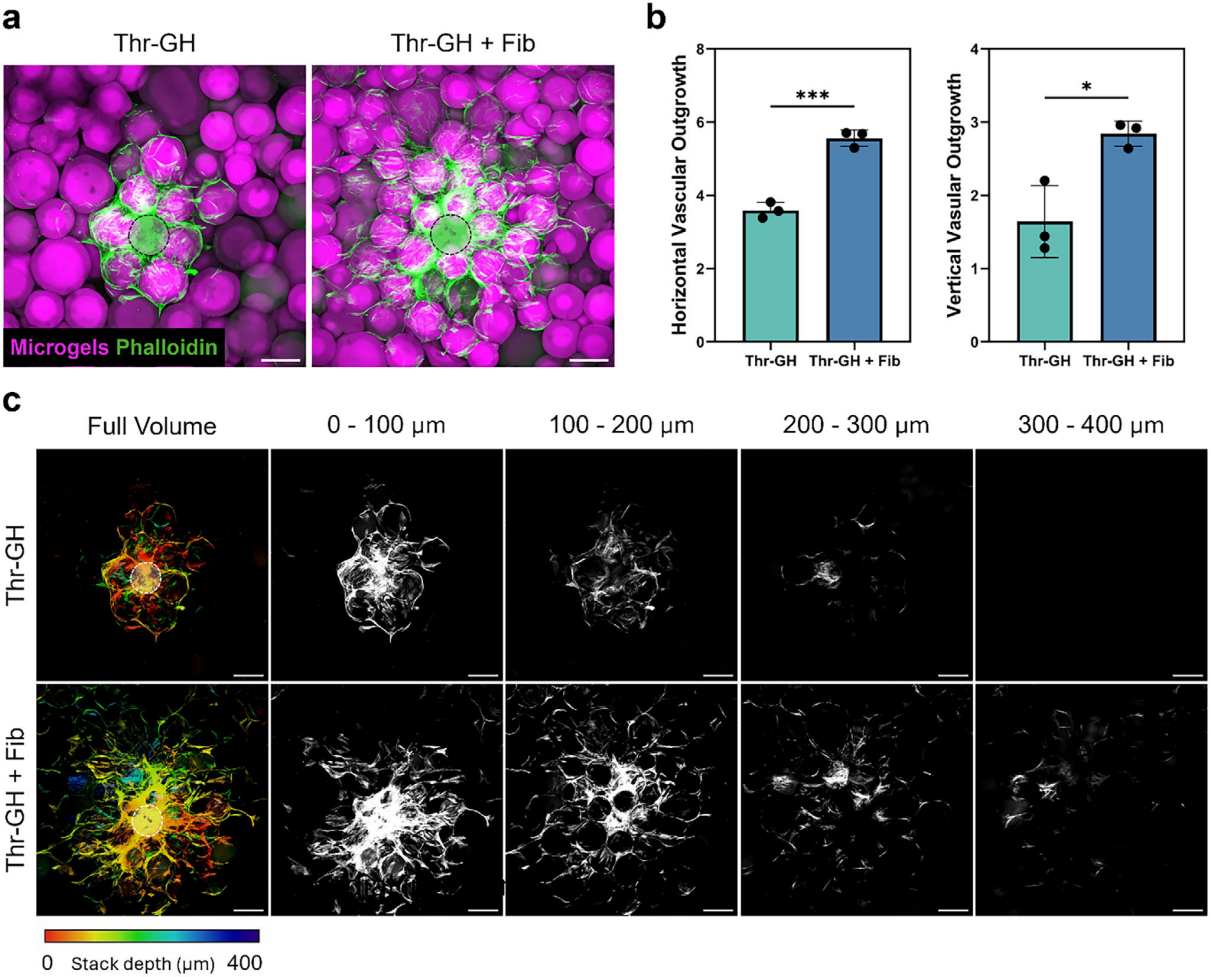
Vascular spheroid sprouting assay. a) Representative maximum intensity projection images of HUVEC‐hMSC spheroids cultured within Thr‐GH and Thr‐GH + Fib for 3 days. Thr‐GHs were made using rhodamine labelled GelMA (shown in magenta), while the spheroids were stained for F‐actin filaments (phalloidin, shown in green). The original position of the vascular spheroids was labelled with black dashed circles. Scale bar: 200 µm. b) Quantification of the horizontal and vertical outgrowth in Thr‐GH and Thr‐GH + Fib, with the average radii of the day 3 vascular networks normalized to a day 0 spheroid. Data are presented as mean ± standard deviation, with a sample size of n = 3. Statistical analysis was performed using an unpaired t‐test with Welch's correction. ^***^ *p* < 0.001, ^*^ *p* < 0.05. c) Representative z‐projections of vascular spheroid sprouting in Thr‐GH and Thr‐GH + Fib, generated from phalloidin‐stained samples. The full volume is shown color‐coded by z‐height, and the original position of the vascular spheroids was labelled with white dashed circles, while 100 µm thick sections are shown in greyscale at different depths through the samples. Scale bar: 200 µm.

We next sought to model how the presence of interstitial fibrin would support the formation of sprouting vascular networks. We generated multicellular vascular spheroids containing a mixture of HUVECs and human mesenchymal stem cells (hMSCs) and assessed their viability using LIVE/DEAD staining (Figure S22a). These vascular spheroids were embedded within Thr‐GH, which was then treated with fibrinogen and cultured for 3 days with vascular endothelial growth factor stimulation for the final 2 days. Confocal microscopy performed at day 3 showed that the Thr‐GH + Fib composite effectively supported vascular sprouting and invasion from the spheroids into the interstitial fibrin network (Figure 6a). We observed substantially less vascular sprouting without the addition of fibrinogen (i.e., the Thr‐GH control), with the spheroid cells primarily remaining adhered to the surface of the microgels. The size of the vascular network was compared to a day 0 spheroid, which revealed substantial horizontal and vertical outgrowth for the Thr‐GH + Fib composite (5.6 ± 0.2 fold; 2.8 ± 0.1 fold, respectively). These values were significantly greater than the corresponding outgrowth observed for Thr‐GH (3.6 ± 0.2 fold; 1.6 ± 0.4 fold, respectively) (Figure 6b). This difference in vascular outgrowth was visualized by re‐slicing the volumetric images (Figure 6c). For comparison, vascular spheroids embedded into imprinted cavities on the surface of bulk GelMA hydrogels exhibited limited invasion into the hydrogel interior, with horizontal and vertical outgrowths of 1.5 ± 0.3 fold and 0.5 ± 0.1 fold, respectively (Figure S22b,c). Together, these findings underscore the crucial role of the interstitial fibrin in facilitating the invasion of a vascular network into the granular hydrogel. This emphasizes the potential of coagulative granular hydrogels to effectively support cellular integration and promote endogenous tissue repair.

**FIGURE 7:**
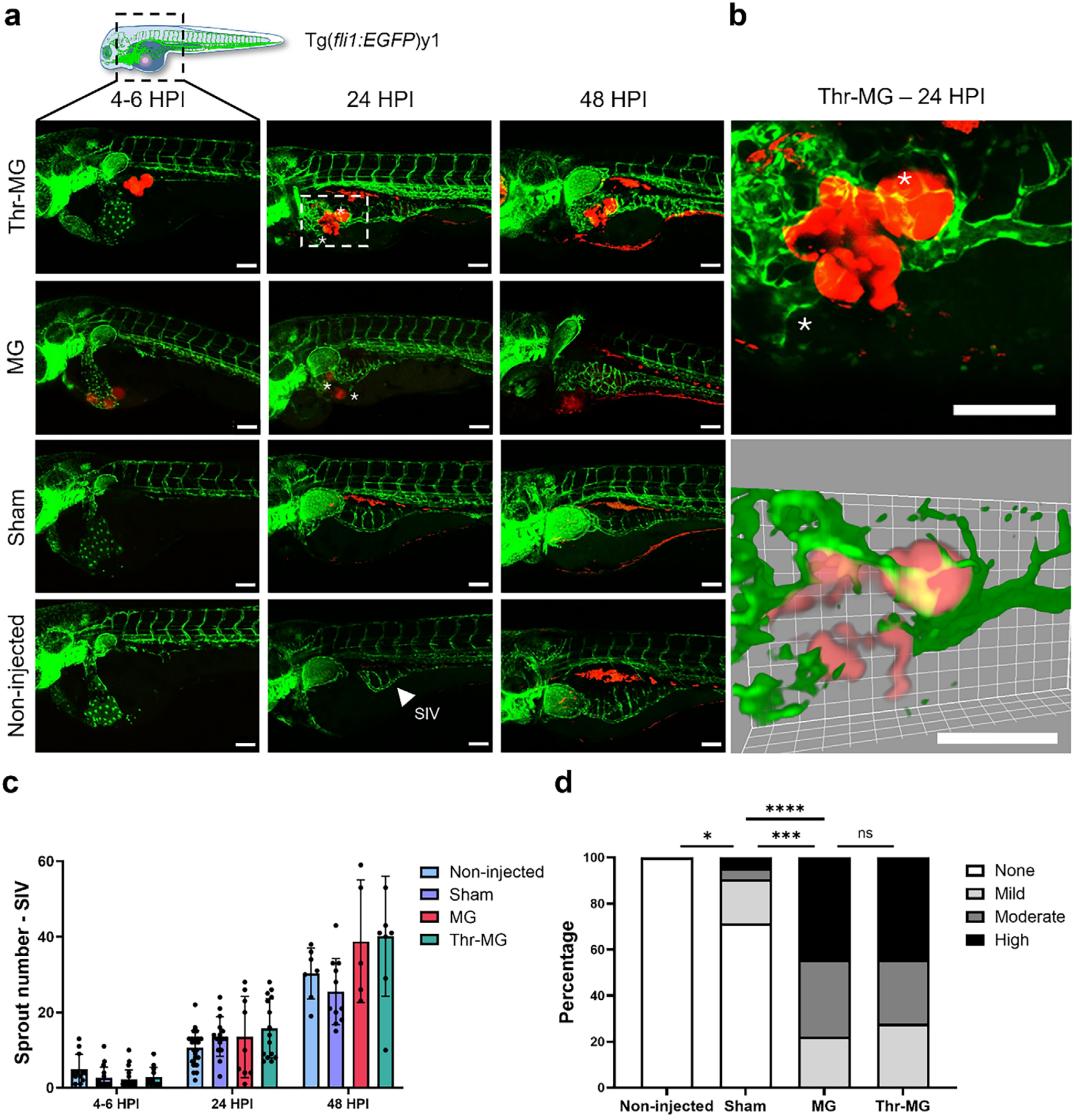
Evaluation of the pro‐angiogenic effect of Thr‐MG and MG in an in vivo zebrafish model. a) A schematic illustrating rhodamine‐tagged microgels (Thr‐MG or MG) injected into the embryo of Tg(*fli1:EGFP*)y1 (a fluorescent reporter line for endothelial cells). Representative confocal fluorescent microscopy images of embryos injected with Thr‐MG or MG, as well as a sham control and a non‐injected control. Images acquired at 4–6, 24, 48 h post‐injection (HPI) show evidence of angiogenesis with new blood vessels sprouting from the sub‐intestinal vessel (SIV). Asterisks in the micrographs of MG and Thr‐MG highlight ectopic angiogenesis. Scale bar: 100 µm. b) A high magnification confocal fluorescence micrograph and 3D rendered image of an embryo injected with Thr‐MG, highlighting ectopic angiogenesis and blood vessels around the biomaterial at 24 HPI. Scale bar: 100 µm. c) Quantification of the number of sprouts from the SIV of the four tested groups at different timepoints (4–6, 24, 48 HPI). Data are presented as mean ± standard deviation, with sample sizes ranging from n = 5 to 23, where each point represents an individual larva. d) A score was attributed to each of the four tested groups (non‐injected, sham, MG, Thr‐MG) to quantify the ectopic angiogenesis at 24 HPI. Score 1 = none; 2 = mild; 3 = moderate; 4 = high. The distribution of scores among the injected embryos is displayed as a stacked bar plot. Statistical analysis was performed using a Fisher's exact test.

We followed up these in vitro observations by conducting a study in Tg(fli1:EGFP) transgenic zebrafish, which express a green fluorescence protein reporter in vascular endothelial cells that enables dynamic visualization of angiogenesis. The diameter of the microgels used in this study was reduced to <100 µm to allow loading into a fine gauge needle (Figure S23), with microgels injected close to the pericardial cavity of embryos at 2 days post fertilization (dpf). The embryos were imaged using confocal fluorescence microscopy at 4–6, 24, and 48 h post‐injection (HPI) for the Thr‐MG and MG groups, as well as non‐injected and sham controls (injected with PBS) (Figure 7a,b). These images, together with live imaging videos (Videos S2 and S3), suggest that host endothelial cells generate a complex vascular network around the injected biomaterial. The number of sprouts of the sub‐intestinal vessel (SIV) was found to increase in number across all the test and control groups over the 48‐h period (Figure 7c). The degree of ectopic angiogenesis at 24 h was scored independently by two observers, assigning a value of either 1 (none), 2 (mild), 3 (moderate), or 4 (high) (Figure 7d). This evaluation showed no significant difference in score between the Thr‐MG (3.2 ± 0.8) and the MG (3.2 ± 0.8) groups; however, the significantly lower value for the sham control (1.4 ± 0.8) confirmed that ectopic angiogenesis was driven largely by the biomaterial rather than the injury caused by injection.

**FIGURE 8:**
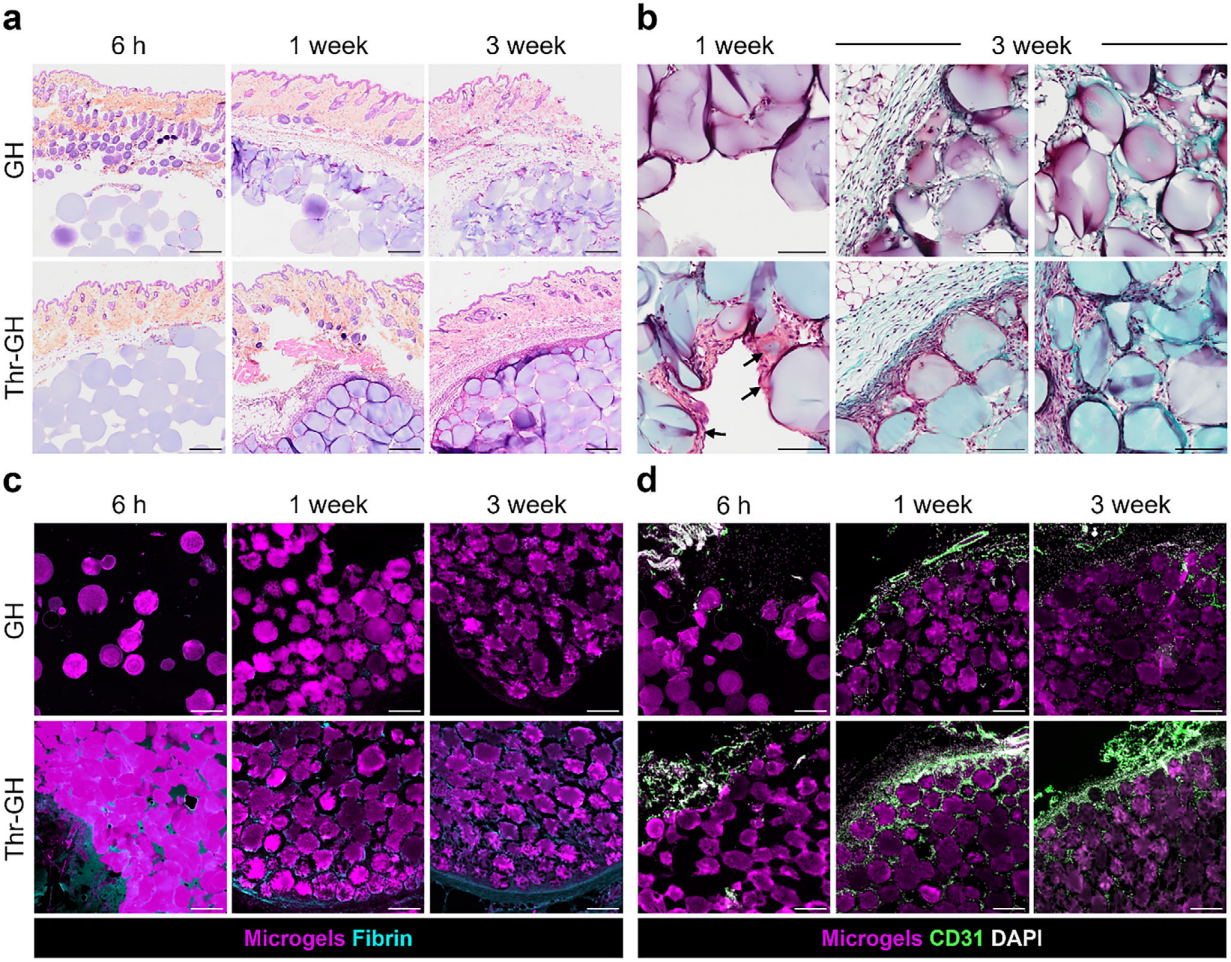
In vivo response to Thr‐GH and GH following subcutaneous injection in mice. The biomaterial and surrounding tissue were explanted at 6 h, 1 week, and 3 weeks post‐injection for histology and immunostaining. a) Representative brightfield images of sections stained with haematoxylin and eosin showing the Thr‐GH retained in a compact form over 3 weeks, with the GH control exhibiting a more disordered structure. Scale bar: 250 µm. b) Representative brightfield images of sections stained with Masson's Trichrome showing the presence of fibrin fibers at 1 week in the Thr‐GH explant (red fibers, marked by black arrow) and collagen deposition at 3 weeks for both granular hydrogel groups. Scale bar: 100 µm. c) Representative fluorescence images of sections immunostained for fibrin (cyan) showing the presence of fibrin for the Thr‐GH explants across all timepoints. Scale bar: 250 µm. d) Representative fluorescent images of sections immunostained for CD31 (green) and nuclear counterstain of DAPI (white). This suggests an increased presence of endothelial cells in the Thr‐GH, compared to the GH control. Scale bar: 250 µm.

We then assessed the in vivo tissue response following subcutaneous injection of Thr‐GH in mice, with unfunctionalized GH as a control. All animals survived the surgery, and wound healing progressed without complications during the monitoring period. The granular hydrogel and surrounding tissue were explanted at 6 h, 1 week, and 3 weeks. Qualitative observations at explantation were that the Thr‐GH samples maintained a cohesive bulk structure and could be readily identified, whereas the GH control had spread laterally over a larger surface area, making localization difficult. This gross observation correlated with haematoxylin and eosin staining, in which the GH samples appeared loosely packed with empty voids at 6 h and 1 week, and inconsistent packing at 3 weeks. In contrast, Thr‐GH demonstrated improved packing with minimal interstitial spaces at all timepoints (Figure 8a). Cell infiltration was evident in both groups, though Thr‐GH showed greater cellular recruitment and attachment by 1 week. After 3 weeks, the GH samples exhibited marked degradation, with irregular microgel morphology in the core, while Thr‐GH remained structurally intact.

Fibrin was present surrounding the Thr‐GH biomaterial, detected as red fibers by Masson's Trichrome staining (Figure 8b). This was confirmed by immunostaining, which showed the presence of fibrin around and between Thr‐GH microgels across all timepoints, with the strongest signal at 6 h (Figure 8c). In the GH control, fibrin was largely absent, with some sparse staining at 1 week attributed to endogenous thrombin catalysis. These results align with our in vitro findings and suggest that the surface‐bound thrombin is capable of catalyzing fibrin formation in vivo. Immunostaining revealed CD31‐positive cells throughout the interstitial voids of Thr‐GH, attributed to endothelial cells that had migrated into the biomaterial (Figure 8d). There were limited CD31‐positive cells in the GH control. Finally, new collagen deposition was evident in both the Thr‐GH and GH groups, detected as light blue fibers by Masson's Trichrome staining (Figure 8b). Collagen was found surrounding the granular hydrogels and within the interstitial spaces between microgels, indicating the start of matrix remodeling. Collectively, these results indicate that following subcutaneous injection, Thr‐GH is capable of catalyzing the formation of a fibrin network to improve stability, reduce microgel degradation, and facilitate endothelial cell infiltration.

## 3 Conclusions

In this study, we have developed an injectable coagulative granular hydrogel using GelMA microgels functionalized with thrombin. We demonstrated that surface‐immobilized thrombin can effectively catalyze fibrinogen into fibrin, resulting in the in situ formation of a secondary fibrin network. The fibrin network acts to “glue” the microgels together, with the granular hydrogel/fibrin composite exhibiting shear and compressive mechanical properties exceeding the bulk GelMA hydrogels. Furthermore, the microporous granular structure and the interstitial fibrin network were shown to support endothelial cell adhesion, proliferation, and invasion. In vivo subcutaneous injection in mice revealed that Thr‐GH maintained structural integrity, supported fibrin deposition, enhanced cell infiltration, and supported collagen remodeling.

Overall, coagulative granular hydrogels address the limitations of existing stabilization strategies by combining their individual advantages while minimizing associated drawbacks. Specifically, the use of thrombin‐functionalized microgels to catalyze a fibrin network provides a biomaterial system with (i) retained injectability, (ii) a method to stabilize the compressive and shear mechanics of the granular hydrogel to the level of the corresponding bulk hydrogel, and (iii) a reinforcing and cell‐compatible secondary network that is formed without the need for any exogenous crosslinkers or external activation (e.g., light). Nevertheless, we have identified limitations to this study, which will be addressed in future work. First, while the catalytic rate can be tuned by modifying the quantity of surface‐bound thrombin, the granular hydrogel structure and mechanics will be dictated by the level of fibrinogen at the site of repair. The concentration of blood plasma fibrinogen ranges from 2–4 mg mL^−1^, with baseline levels varying based on the genetics, sex, and age of an individual, and dynamically upregulated in infection and acute inflammation (2–3 fold) [48]. A second limitation of our study is the lack of immune cell profiling in our in vivo study. Granular hydrogels have been shown to lead to favorable inflammatory responses and reduced fibrotic capsule formation compared to bulk hydrogels [49]; however, it would be important to assess the impact of the fibrin network over long time periods. A third limitation is the low throughput of the microfluidics used in this work: while this technique yielded uniform microgel dimensions, it is not scalable for large‐scale production. Clinical translation would require adaptation to a more scalable microgel fabrication method, with additional controls over pathogens, endotoxins, and other contaminants. The absence of any additional exogenous crosslinkers or photo‐annealing in our approach is favorable from a translational standpoint, as is the fact that sealants containing fibrinogen and thrombin have already gained regulatory approval (e.g., Tisseel, Evicel). The methods used in this work are adaptable to other granular hydrogel systems, provided there are chemically addressable moieties on the microgel surface, and future work will look to expand this technology to different biomaterials and validate the results from this study in different tissue repair scenarios.

## 4 Experimental Section

### 4.1 Gelatin Methacryloyl (GelMA) Synthesis

GelMA was synthesized as previously reported [36]. Type A gelatin from porcine skin (300 g Bloom, Merck G1890) was dissolved to 100 mg mL^−1^ in ultrapure water (18.2 MΩ cm) under stirring at 50°C. Methacrylic anhydride (Merck 276685) was then added to the solution at a ratio of 0.6 mL per gram of gelatin. The solution was stirred vigorously for 3 h and then centrifuged at 3500 g for 3 min at room temperature. The supernatant was collected, diluted with 50 mL of pre‐warmed ultrapure water (∼40°C), and then transferred into a 12–14 kDa regenerated cellulose dialysis tubing (Fisher Scientific 11465859). The solution was dialyzed against 5 L ultrapure water at 40°C for 6 days with the water changed twice a day. After dialysis, the pH of the solution was adjusted to 7.4 with 1 m sodium bicarbonate (VWR RC‐091) and sterile‐filtered with a Corning vacuum filter system (0.22 µm, Scientific Laboratory Supplies 430769). The GelMA solution was then freeze‐dried using a Benchtop Pro 3L ES‐S5 freeze dryer (SP Scientific) and stored at −20°C for later use.

### 4.2 GelMA Characterization

^1^H‐NMR spectroscopy was performed to confirm the conjugation of methacrylate groups. 20 mg of GelMA and gelatin were dissolved separately in 0.75 mL deuterium oxide (VMR 1.13366). 700 µL of each sample was loaded into NMR tubes (Norell NORS55007), with ^1^H‐NMR spectra obtained using a JEOL ECZ 400 MHz NMR spectrometer. Fluoraldehyde *o*‐Phthaldialdehyde Reagent Solution (Fisher Scientific 10530054) was used to quantify the degree of functionalization of each GelMA batch. Gelatin standards of 1, 0.5, 0.2, 0.1, 0.05, 0.02 mg mL^−1^ were prepared in PBS (Fisher Scientific 10388739). Each batch of GelMA was dissolved in PBS to a concentration of 1 mg mL^−1^. 300 µL of each sample/standard was mixed with 600 µL fluoraldehyde reagent solution. Upon mixing, 250 µL triplicates of each sample/standards mix was transferred to individual wells of a black 96‐well plate (Greiner Bio‐One 655090). Fluorescence was measured (λ_ex_ = 360 nm, λ_em_ = 450 nm) using aSynergy Neo2 microplate reader (BioTek).

### 4.3 Microfluidic Chip Fabrication

Molds for T‐junction microfluidic chips were designed on Solidworks (Dassault Systèmes) and printed using a Form 3B+ SLA 3D printer (Formlabs). After removing the printing supports, the 3D printed molds were cured at 60°C for 30 min. Polydimethylsiloxane (PDMS) was made by mixing silicone elastomer base and curing agent from the Silicone Sylgard 184 Kit (Scientific Laboratory Supplies 63416.5S) at a 10:1 mass ratio, which were then poured into the 3D printed molds and baked at 60°C for 4 h. Fully cured PDMS chips were removed from the mold and cleaned with ethanol. The PDMS chips were plasma‐treated using a FEMTO low‐pressure plasma cleaner (Diener Electronics) for 30 s under 100 W and 1 bar O_2_, and then immediately sealed with a plasma‐treated chip base to create water‐tight microchannels. This assembly was then baked in the oven at 60°C for 1 h to enhance sealing between the PDMS and the base. The chip design can be found in the Supplementary Data (Figure S3).

### 4.4 Microgel Fabrication

A 5 mL Terumo syringe containing a solution of 80 mg mL^−1^ uncrosslinked GelMA and 2.5 mg mL^−1^ LAP (Merck 900889) in PBS was loaded onto an AL1010 syringe pump (World Precision Instruments). A second syringe containing mineral oil (Merck M8410) with 5% v/v Span 80 (Merck S6760) was loaded onto an identical syringe pump. The flow rates of the GelMA/LAP solution and the mineral oil were set to 3 and 30 µL min^−1^, respectively. The two solutions were flowed into the microfluidic chip via low‐density polyethylene tubing (Fisher Scientific 13190873). Once a continuous flow of monodisperse microdroplets was observed by eye in the outlet tubing, the microdroplets were crosslinked in one of two ways. In situ *crosslinking* was performed inside the outlet tubing with a UV lamp (365 nm, ∼19 mW cm^−2^, Vilber), with the resulting microgels centrifuged (2000 g, 2 min, room temperature) and washed with 0.1% v/v Tween‐20 (Fisher Scientific 10066100) in PBS and then PBS alone to remove the oil phase before storing at 4°C. *Crosslinking after washing* was performed by transferring uncrosslinked microdroplets to 15 mL Falcon tubes, which were cooled to −20°C, then centrifuged at 2000 g for 2 min. The supernatant was removed and replaced with mineral oil. The washing process was repeated, with the microdroplets resuspended first in mineral oil, then in PBS, and finally in PBS with 2.5 mg mL^−1^ LAP. The microdroplets were crosslinked in a trough on an ice bath for 1 h under a UV lamp. The microgels were collected and washed with PBS a further 3 times before storing at 4°C. The latter protocol was used for the gelation test (brightfield microscopy and SEM), while the former protocol was used for all other experiments.

### 4.5 Fluorescent Microgel Fabrication

GelMA was dissolved in PBS to a concentration of 20 mg mL^−1^. NHS‐rhodamine (Thermo Fisher Scientific 46406) was added to the solution at a ratio of 30 mg per gram of GelMA. The solution was stirred vigorously for 5 h at 50°C in the dark. After the reaction, the solution was transferred into a 12–14 kDa regenerated cellulose dialysis tubing and dialyzed against 5 L ultrapure water at 40°C for 6 days with water changed twice a day. After dialysis, the pH of the solution was adjusted to 7.4 with 1 M sodium bicarbonate and sterile‐filtered with a 0.22 µm Corning vacuum filter system. The rhodamine‐tagged GelMA was freeze‐dried and stored at ‐20°C for later use. Fluorescent GelMA microgels were prepared and functionalized as above, but using a 1:9 mixture of rhodamine‐GelMA and GelMA instead of pure GelMA.

### 4.6 Size Analysis

The diameter of microgels in oil and PBS was measured using ImageJ (Fiji) (n *>* 300). The threshold of the images was adjusted to reveal the outlines of the microgels, then the ‘analyze particle’ function was used to measure the Feret's diameter of the microgels. The size distributions were plotted as average shifted histograms as described elsewhere [50].

### 4.7 Thrombin Functionalization

Microgels were centrifuged at 2000 g for 2 min, with the pellet re‐suspended in 0.1 M (2‐(*N*‐morpholino)ethanesulfonic acid) (MES) buffer at pH 5.5 (Fisher Scientific 15442958). This process was repeated four times before a 250 µL pellet (equivalent to 20 mg of freeze‐dried microgels) was suspended in 3 mL of MES buffer. 1 mL of 40 mg mL^−1^ EDC (Merck 341006) and 1 mL of 65 mg mL^−1^ sulfo‐NHS (Merck 56485) were added to the reaction. The suspension was stirred at room temperature for 30 min before removing the supernatant. The activated microgels were then resuspended in 3 mL of PBS and 50 µL of 100 U mL^−1^ thrombin (Merck T1063) and stirred at room temperature overnight. After the reaction, thrombin‐functionalized microgels (Thr‐MG) were washed with PBS (5 times, 2 min, 2000 g) to remove any unbound thrombin and reaction byproducts. Microgel controls were prepared as above, but without the addition of thrombin (EDC‐activated microgels, EDC‐MG) or with the addition of 1 mL of 10 mg mL^−1^ bovine serum albumin (Merck A2153) in PBS instead of thrombin (BSA‐functionalized microgels, BSA‐MG). All microgels were stored in PBS at 4°C until further use.

### 4.8 Thrombin Activity Assay

The enzymatic activity of the Thr‐MG, the microgel controls, and the supernatant from the functionalization process was measured using a thrombin activity fluorometric assay kit (Merck MAK242), performed according to the manufacturer's protocol. Briefly, 50 µL of each diluted sample was added to a white 96‐well plate (Greiner Bio‐One 655207), alongside 50 µL thrombin standards (0, 5, 10, 15, 20, 25, 50, 100, 200, 300 ng per well). Each sample/standard was mixed with 50 µL of substrate mix. Fluorescence signals were measured (λ_ex_ = 350 nm, λ_em_ = 450 nm) every 5 min for 60 min using a microplate reader at 37°C. Based on the plotted data, two time points from the linear regions of samples and standards were chosen to calculate the difference in fluorescence (ΔRFU) of each. The standard curve was plotted as concentration of thrombin standards vs ΔRFU, which was used to determine the thrombin concentration of the samples. Thrombin activity in individual batches of Thr‐MG was assessed when freshly prepared (n = 6) and after storage at 4°C for 1 week (n = 5) and 2 weeks (n = 6).

### 4.9 Imaging of Microgel‐Fibrin Network

Fibrinogen from human plasma (Thermo Fisher Scientific F3879) was dissolved in phenol‐free Dulbecco's Modified Eagle Medium/Nutrient Mixture F‐12 (DMEM/F‐12, Thermo Fisher Scientific 21041025) to a concentration of 50 mg mL^−1^. 150 µL of either Thr‐MG, EDC‐MG, or BSA‐MG pellets were resuspended in 400 µL of DMEM/F‐12 (equivalent to a microgel concentration of 30 mg mL^−1^) and then mixed with 250 µL of fibrinogen solution in a 4‐well plate (Thermo Fisher Scientific 176740). A fibrin gel control was prepared the same way by mixing 400 µL of a 2.5 U mL^−1^ thrombin solution with 250 µL of fibrinogen solution. The well plate was sealed with parafilm and placed on an orbital shaker at 50 rpm and 37°C. The morphology of the microgel samples was observed at different reaction intervals (0, 2, 7, 24 h) using brightfield microscopy (MOTIC S3). These samples were also fixed for SEM using 2.5% v/v glutaraldehyde in 0.1 M sodium cacodylate buffer (pH 7.4) for 2 h. The samples were rinsed twice with ultrapure water and then dehydrated by 5 min immersions in 30, 50, 70, 80, 90, 99.8, and 100% v/v ethanol. Dehydrated samples were dried using a Leica CPD300 critical point dryer, then sputter‐coated with gold (∼12.5 nm) using a Quorum Emitech K575X metal sputter coater at 40 mA sputter current for 60 s before imaging using an FEI Quanta 200 scanning electron microscope. Confocal fluorescence microscopy was also used to visualize the microgel‐fibrin network. Fluorescent Thr‐MGs and BSA‐MGs were resuspended in DMEM/F‐12. 50 µL of each suspension was mixed with 100 µL of a 20 mg mL^−1^ fluorescent fibrinogen solution in separate wells of an 8‐well uncoated µ‐slide (Thistle Scientific 80801). Fluorescent fibrinogen solution was prepared by dissolving 5 mg of Alexa Fluor 488‐tagged fibrinogen (Thermo Fisher Scientific F13191) and 45 mg of fibrinogen in 2.5 mL PBS. After incubation at 37°C overnight, the samples were imaged using multi‐laser confocal laser scanning microscope (Leica SP5II and Leica SP8), equipped with a 488 nm argon laser and 561 nm solid state yellow laser. Images were acquired using 10X and 20X dry objective lenses with a step size of 4.28 µm used for 3D stacks. Images were exported using LAS X software (Leica), processed using ImageJ (Fiji), and reconstructed in 3D using ImarisViewer.

### 4.10 Formation of Jammed Granular Hydrogels

Jammed granular hydrogels (GH, Thr‐GH) were made by centrifuging microgel suspensions at 2000 g for 2 min in either 15 mL Falcon tubes using an Eppendorf centrifuge 5702 or a 48‐well plate using a SIGMA 4–16K centrifuge. After centrifugation, the supernatant was removed.

### 4.11 Light Sheet Microscopy of Composite Granular Hydrogels

Thr‐GH made from rhodamine‐tagged Thr‐MG was transferred to cryomolds (Agar Scientific AGG4581) before the addition of either 20 mg mL^−1^ Alexa Fluor 488‐tagged fibrinogen solution in PBS or PBS alone (no fibrinogen control). After overnight incubation at 37°C, the two samples (Thr‐GH ± Fib) were removed from the mold (10 × 10 × 5 mm) and glued onto a sample holder. The sample holder was inserted into the imaging chamber of a Zeiss Z.1 light sheet fluorescence lamp. The imaging chamber was filled with PBS, and images were acquired with a z‐step size of 1.23 µm using 488 and 561 nm argon lasers and a 5X dry objective lens. The images were 3D rendered using arivis Vision4D software. The void area in the granular hydrogels was segmented from the acquired image stacks. Packing density was calculated as the total stack volume minus the void fraction, all divided by the total stack volume.

### 4.12 Injectability Test

Thr‐GH and unfunctionalized granular hydrogels (GH) were loaded into 2.5 mL Terumo syringes fitted with 26‐gauge needles (Fisher Scientific 15301557) and extruded manually. An extruded sample was prepared on a glass coverslip and imaged using brightfield microscopy.

### 4.13 Granular Hydrogel Stability Test

Thr‐GH and Thr‐GH + Fib (∼250 µL each) were prepared in Eppendorf tubes as previously described, with 1 mL of PBS added on top. The tubes were placed in an Eppendorf ThermoMixer C at 23°C with continuous agitation at 500 rpm. Images were taken at defined time points to monitor changes in integrity over time.

### 4.14 Rheology

All rheological measurements (n = 3) were performed using an Anton Parr Physica MCR301 rheometer equipped with a 12.5 mm diameter parallel plate geometry. Measurements were performed with samples loaded in a sandblasted 24‐well plate (Greiner Bio‐One 662160) attached to the plate at 37°C. Four granular hydrogel groups were tested (Thr‐GH ± fibrinogen, GH ± fibrinogen). These were centrifuged at 2000 g for 2 min, and then transferred into the well plate (∼500 µL per well). The granular hydrogel groups with the addition of fibrinogen in the well plate were incubated at ∼37°C overnight before measurement. Bulk hydrogel controls were formed and crosslinked inside the well plate: 80 mg mL^−1^ GelMA in PBS with 2.5 mg mL^−1^ LAP was crosslinked using a UV lamp (365 nm, ∼6.8 mW cm^−2^, Spectroline ENF‐260C/F) for 10 min and bulk fibrin gels were formed by mixing 490 µL of 10 mg mL^−1^ fibrinogen solution with 10 µL of 1 U mL^−1^ thrombin solution. Oscillatory stain sweeps were performed from 0.1%–1000% strain at 10 rad s^−1^. The plate was lowered until the full surface of the gel was probed (∼2.7 mm thickness). The oscillatory frequency sweeps were run at 1–100 rad s^−1^ under 0.1% strain. Viscosity measurements were measured over a rotational shear rate of 0.01–100 s^−1^. Shear ramp tests were set to measure the shear moduli under alternating low strain (0.1% strain) and high strain (200%) at a constant frequency of 10 rad s^−1^ (2.5 cycles, 110 s per cycle). Time sweeps were measured overnight at constant frequency (10 rad s^−1^) and strain (0.1%).

### 4.15 Compression Testing using a Dynamic Mechanical Analyzer

Dynamic cyclic compression testing was performed using a DMA850 (TA Instruments) fitted with a submersion compression clamp at 37°C. MG and Thr‐MG in complete endothelial cell media were jammed into granular hydrogels (GH, Thr‐GH), and loaded into a 2.5 mL Terumo Syringe. ∼80 µL of the granular hydrogel samples were extruded into separate cylindrical molds with a diameter of 6 mm and height of 3 mm. 20 µL of 10 mg mL^−1^ fibrinogen in endothelial cell medium was added to the granular hydrogels, and samples were incubated overnight at 37°C. Bulk GelMA controls were prepared using a similar protocol, with 80 mg mL^−1^ GelMA precursor and 2.5 mg mL^−1^ LAP in complete endothelial cell media added to the mold, crosslinked using UV light (∼6.8 mW cm^−2^, 10 min). Calipers were used to measure the dimensions of the samples before they were loaded onto the testing rig and secured with a clamp at a preload of 0.01 N. The chamber was then filled with ∼4 mL complete endothelial cell media to fully immerse the sample. Oscillation strain sweeps were performed after a 3 min soaking period to reach equilibrium under force control with 1% strain increments at 1 Hz and a force track of 115%. The force track feature was enabled to maintain the ratio between static and dynamic forces, ensuring that the static force scaled with the stiffness of the samples. This adjustment prevented overstressing samples during cyclic loading. While the force track feature facilitated dynamic adjustments for consistent amplitude without overstraining, it did not directly control the thickness or modulus. The thickness of the samples was also tracked during the tests. Initially, a sweep from 0.01–60% strain was conducted to determine the failure strain of each sample. Subsequent sweeps were performed from 0.01–25% for GelMA and Thr‐GH + Fib, and from 0.01–15% for GH + Fib (these samples could not reach 25% strain). Dynamic compressive moduli were calculated using the gradient of the stress‐strain plot between 2–7% strain. To evaluate the cyclic performance of Thr‐GH + Fib, the sweeps from 0.01–25% strain were performed for 30 cycles.

### 4.16 Compression Testing using a Mechanical Tester

GH + Fib and Thr‐GH + Fib samples were prepared as described above. Callipers were used to measure the precise dimensions of each sample before and after testing. Compression tests were conducted at ambient temperature and humidity using a Starrett FMS500 machine with a 100 N load cell, at a rate of 0.5 mm min^−1^, until a final displacement of 0.33 mm was reached (∼10% strain). Samples were placed in individual wells of a 24‐well plate and incubated at 37°C between tests, either for 10 consecutive days (daily testing) or a single 10‐day incubation. Compressive moduli were calculated from the gradient of the stress‐strain curve between 2–7% strain based on the linear region of the curves.

### 4.17 Infiltration Test

Thr‐GH were loaded into 1 mL Terumo syringes with narrow tips removed (∼150 µL each). Half of the syringes were then topped with 20 µL of 10 mg mL^−1^ fibrinogen in PBS. Similarly, GelMA bulk hydrogels (175 µL of 80 mg mL^−1^ GelMA in PBS with 2.5 mg mL^−1^ LAP) were formed by UV crosslinking (∼6.8 mW cm^−2^, 10 min) inside precut 1 mL syringes (∼10 mm thickness). After gel formation, 50 µL of one of the following dye solutions was added to each syringe: 1 mg mL^−1^ rhodamine B (Merck R6626), 2.5 mg mL^−1^ FITC‐dextran 40 kDa (Merck FD40), 25 mg mL^−1^ FITC‐dextran 150 kDa (Merck 46946), all prepared in PBS. Dye infiltration was monitored over time using a camera. An additional 50 µL of dye solution was added to each sample after ∼10 h to prevent drying.

### 4.18 Passive Dedradation Test

GH and Thr‐GH samples (∼250 µL each) were prepared in pre‐weighed Eppendorf tubes in triplicate. An additional three tubes of Thr‐GH were resuspended in 300 µL of 10 mg mL^−1^ fibrinogen in PBS, centrifuged, and the supernatant was removed. These samples were then incubated at 37°C for 24 h to form Thr‐GH + Fib. All tubes were topped up to ∼1 mL with 0.2 mg mL^−1^ sodium azide in PBS and weighed again. Tubes were sealed with parafilm and incubated at 37°C for passive degradation. Sample masses were recorded, and media were exchanged (500 µL per tube) daily for the first 3 days and every 2 days thereafter, for a total of 31 days. Hydrogel integrity was assessed at each time point by inverting the tubes and capturing images with a camera.

### 4.19 Cell Culture

Human umbilical vein endothelial cells (HUVECs, Merck SCCE001) were cultured using complete endothelial cell media consisting of 5% v/v fetal bovine serum, 1% v/v endothelial cell growth supplement, and 1% v/v penicillin‐streptomycin in endothelial cell basal medium (Caltag Medsystems SC‐1001). Human mesenchymal stem cells from bone marrow (hMSCs, Merck C12974) were cultured using low glucose DMEM (Thermo Fisher Scientific 21885108) supplemented with 10% v/v fetal bovine serum (Merck F9665), 1% v/v penicillin‐streptomycin (Fisher Scientific 11548876), 5 ng mL^−1^ recombinant human fibroblast growth factor basic (PeproTech 100–18B). All cells were cultured in T‐75 flasks (Greiner Bio‐One 658175), with the HUVEC culture flasks pre‐coated with fibronectin (Merck F1056) at ∼2 µg cm^2^. The cell culture media were changed every two days and passaged at a confluency of 80% using 0.05% v/v trypsin‐EDTA (Merck T3924) in PBS. All cells were used at passage nine or lower. All microgels used for cell culture were sterilized under UV (254 nm, ∼0.7 mW cm^−2^, Spectroline ENF‐260C/FE) for 1 h, while the thrombin functionalization was performed as described above, but under sterile conditions. All chemical reagents were either sterile‐filtered (GelMA, LAP, EDC, sulfo‐NHS, MES buffer, fibrinogen), autoclaved (agarose), or prepared under sterile conditions (thrombin, PBS).

### 4.20 Imaging Cell Attachment and Proliferation

10 mg mL^−1^ ultrapure agarose in PBS (Fisher Scientific 16550100) was prepared, sterilized, and reheated in a microwave (HADEN 193926) for 60 s. 500 µL of warm agarose solution was added to separate wells of a 48‐well plate and gelled at 4°C for 1 h. This was to ensure that the samples were close to the surface of the well to be imaged under an upright multiphoton microscope. Sterile, rhodamine‐tagged microgels (MG or Thr‐MG) were jammed into granular hydrogels on top of the agarose gel in the 48‐well plate (∼150 µL per well, ∼1.6 mm thickness). The granular hydrogels were then incubated overnight in 50 µL of complete endothelial cell media either supplemented with 10 mg mL^−1^ fibrinogen (for Thr‐GH + Fib, GH + Fib) or unsupplemented (Thr‐GH). Suspensions of HUVECs (1.7 × 10^5^ cells mL^−1^ for the proliferation experiment and 1 day cell infiltration study, 6.7 × 10^5^ cells mL^−1^ for the 1 day adhesion study) were prepared in complete endothelial cell media, and 300 µL was seeded onto each sample. Half media changes (150 µL) were performed every 2 days, with samples harvested after 1, 4, or 7 days of culture. These samples were fixed using 40 mg mL^−1^ formaldehyde (Merck P6148) in PBS for 15 min and then washed 3 times with PBS. The samples were permeabilized in 0.1% v/v Triton X‐100 (Merck X100) in PBS for 30 min, blocked with 0.3% v/v Triton x‐100 and 5 mg mL^−1^ BSA in PBS for 30 min. The samples were stained through incubation with a 660 nM solution of Alexa Fluor 488 phalloidin (Thermo Fisher Scientific A12379) for 2 h and a 1 µg mL^−1^ solution of 4’,6‐diamidino‐2‐phenylindole dihydrochloride (DAPI, Thermo Fisher Scientific 62248) for 30 min. The samples were then washed 3 times with PBS for 10 min after each step. The stained samples were kept at 4°C before imaging. Stained samples were imaged from the top of the biomaterial using a Leica SP8 AOBS confocal laser scanning microscope attached to a Leica DM6000 upright epifluorescence microscope (multiphoton microscope). Image stacks were acquired with a step size of 3.77 µm using a 10X dry objective lens, a 488 nm argon laser, a 561 nm solid‐state yellow laser, and a 750 nm multiphoton laser. Acquired images were processed using Fiji.

### 4.21 Quantifying Cell Metabolism

An alamarBlue assay (Thermo Fisher Scientific DAL1025) was used to quantify the metabolic activity of the seeded cells over time (n = 3). Samples were prepared as described for the imaging study using non‐fluorescent microgels and 2 × 10^4^ cells per well. After 1, 4, and 7 d in culture, 300 µL of 20% v/v alamarBlue reagent in complete endothelial cell media was added to each well to yield a final alamarBlue reagent concentration of 10% v/v. The samples were incubated at 37°C for 1 h. 400 µL of supernatant was taken from each well and centrifuged in Eppendorf tubes to remove any unbound microgels or cells. 100 µL triplicate of each supernatant was then transferred to a black 96‐well plate. Fluorescence readings were measured (λ_ex_ = 570 nm, λ_em_ = 585 nm) using a microplate reader. The supernatant was replaced with 300 µL fresh cell media, and half media changes (150 µL) were performed every 2 days.

### 4.22 Vascular Spheroid Invasion Assay

HUVECs and hMSCs were harvested from culture flasks with trypsin and counted using an automatic cell counter (Denovix CellDrop FL). The cells were combined at a 2:1 ratio and resuspended in a 2:1 mixture of HUVEC media and hMSCs media to a total cell concentration of 1 × 10^4^ cells mL^−1^. This cell suspension was transferred into ultra‐low attachment 96‐well plates (Merck 7007) with 2000 cells per well. The well plate was tapped gently and cultured for 3 days to form vascular spheroids. The viability of vascular spheroids was assessed using a LIVE/DEAD staining kit (Fisher Scientific L3224) following the manufacturer's protocol. These spheroids were then cultured with the biomaterial (Thr‐GH + Fib, Thr‐GH, bulk GelMA hydrogels) [40]. Briefly, the spheroids were harvested into Eppendorf tubes and then transferred to a black 96‐well plate pretreated with anti‐adherence rinsing solution (Stem Cell Technologies 07010) (∼40 spheroids per well). Sterile Thr‐GHs were transferred into the 96‐well plate (∼100 µL per well, ∼3.1 mm thickness) and gently mixed with spheroids using a pipette tip. 20 µL of 10 mg mL^−1^ fibrinogen in the 2:1 mixed cell media was added to half of the wells to form the Thr‐GH + Fib composite. After 4 h of incubation, 100 µL of the 2:1 mixed media was added to each well. A total of 100 µL of 80 mg mL^−1^ GelMA hydrogel was cast in sterile PDMS molds using the same photoinitiator concentration and UV crosslinking time as previously used to form cylindrical constructs (Ø 11 mm × h 8 mm) with a cavity (Ø 5 mm × h 2.5 mm) on top. The hydrogels were then transferred to individual wells of a 48‐well plate, and spheroids were seeded into the cavities, followed by the addition of 100 µL of 2:1 mixed media. After 1 day of culture, the media was replaced with 100 µL of fresh 2:1 mixed media supplemented with 100 ng mL^−1^ recombinant human vascular endothelial growth factor (Thermo Fisher Scientific PHC9394). The samples were cultured for another 2 days before fixation with 40 mg mL^−1^ formaldehyde in PBS for 40 min and then washed 3 times with PBS. The samples were then permeabilized, blocked, and stained with Alexa Fluor 488 phalloidin and DAPI, as described above, except that phalloidin was stained through incubation overnight at 4°C. Stained samples were imaged from the bottom of the well using a Leica SP8 multi‐laser confocal laser scanning microscope equipped with a 488 nm argon laser and 561 nm solid‐state yellow laser. Images were acquired using 10X dry objective lenses with a step size of 4.28 µm and used to generate 3D stacks. Acquired images were processed using Fiji. Horizontal outgrowth was calculated by normalizing the average radii of the day 3 vascular networks from maximum projection images to the radius of a day 0 spheroid. Vertical outgrowth was calculated using the radius of a day 0 spheroid (r_0_) to normalize the vertical displacement in the day 3 vascular networks (total depth—r_0_).

### 4.23 Zebrafish Study

Zebrafish (*Danio rerio*) were raised and maintained under standard laboratory conditions at 28.5°C with a 14:10 light/dark cycle. Larvae from the *Tg(fli1:EGFP)* transgenic line were used for all experiments. Experiments were approved by the Animal Welfare and Ethical Review Body of the University of Bristol and performed under UK Home Office project licence (PPL PP4700996). At 2 days post‐fertilization (dpf), larvae were anesthetized in tricaine solution (150 mg L^−1^, Merck MS222) and positioned laterally in a 3% w/v agarose injection mold prepared in 90 mm petri dishes. When necessary, embryos were manually dechorionated prior to injection using fine tweezers under a Nikon SMZ 745 stereomicroscope. Glass capillaries (borosilicate glass, Sutter Instrument) were pulled using a Flaming/Brown micropipette puller (Sutter Instrument) to obtain needle tips with a ∼100 µm opening. MG and Thr‐MG were fabricated as described above, but with the GelMA/LAP flow rate adjusted to 1 µL min^−1^ to reduce the microgel diameter to under 100 µm. The needles were pre‐coated with 1% w/v BSA to prevent microgel adherence. Each needle was backfilled with 20 µL of microgel suspension using Microloader pipette tips and mounted on a pneumatic microinjection system (Harvard Apparatus). Injections were performed at 8X magnification under a stereomicroscope. For the experimental group, unfunctionalized MGs or Thr‐MGs were injected close to the pericardial cavity of 2 dpf larvae. Sham controls were injected with PBS using the same needle specifications and handling conditions. Following injection, larvae were transferred to 12‐well plates (1 larva per well) containing 2 mL of embryo medium and maintained at 27 ± 0.5°C. Embryos were monitored for viability, and dead individuals were removed after 2 h. For imaging, live larvae were anesthetized and embedded laterally in 1% w/v low‐melting‐point agarose (Fisher Scientific 16520050). Z‐stacks and time‐lapse images were acquired using a Leica SP5II or SP8 confocal laser scanning microscope. Maximum intensity projections were generated using LAS AF Lite software (Leica) and Image J (Fiji). Video recording was also performed using a ZEISS Celldiscoverer 7 with a 10X objective, capturing the same larvae every 20 min over a 24 h period (supplementary videos were cropped between 7–8 hpi). Additional static imaging was conducted at 24‐h intervals post‐injection.

### 4.24 Mouse Subcutaneous Injection

Thr‐GH and GH were prepared under sterile conditions and stored in 5% v/v penicillin‐streptomycin in PBS. Prior to the surgery, the granular hydrogels were washed with PBS and loaded into 1 mL syringes. 10 BALB/c mice were used for each group (all males, with weights between 21.1–28.8 g, Charles Rivers Laboratories) with samples explanted at 3 timepoints: 6 h post‐injection (n = 2), 1 week post‐injection (n = 4), and 3 weeks post‐injection (n = 4). The surgical procedures were performed by one surgeon in compliance with the British Home Office regulations of the use of laboratory animals and the University of Manchester Animal Welfare and Ethical Committee Legislation under PPL PP6455727. Induction and maintenance of anaesthesia was performed using 2–3% isoflurane (Abbot Laboratories) and 2 L min^−1^ oxygen. While supine, the mice's bilateral inguinal areas were shaved and cleaned using antiseptic betadine solution (1% w/w iodine in aqueous solution, Animalcare Ltd). Prior to injection, the femoral vessels were identified bilaterally. 100 µL of Thr‐GH was injected subcutaneously in the proximity of the right‐side femoral vessels, while 100 µL of GH was injected similarly in the left inguinal area. At the end of the procedure, mice received adequate local anaesthesia with buprenorphine (0.1 mg kg^−1^) and were placed in an incubator for recovery. All animals were monitored throughout the study to ensure their welfare was maintained. Wound healing progressed without complications during the monitoring period. At the 3 different timepoints described above, an incision was made over the sample insertion area. The hydrogels together with surrounding soft tissue were harvested and fixed in 40 mg mL^−1^ formaldehyde in PBS overnight at 4°C. After fixation, the samples were washed twice with PBS for 5 min each and then stored in PBS until further processing.

### 4.25 Explant Processing

Fixed explant samples stored in PBS were cut into two halves with a scalpel. One‐half was submerged in Optimal Cutting Temperature embedding medium (Agar Scientific AGR1180), frozen into a solid block, and cryo‐sectioned at 10 µm thickness. Sections were collected using Superfrost Plus microscope slides (VWR 631‐0108) and kept frozen until use. The other half was dehydrated through graded ethanol (70%, 90%, 100%) and cleared with xylene. The cleared samples were then immersed in molten paraffin wax, embedded in blocks, which were allowed to solidify at room temperature. Paraffin blocks were sectioned at 5 µm using a rotary microtome. Sections were mounted on glass slides and stored at room temperature. Frozen sections were used for immunostaining of CD31 (R&D systems AF3628) and fibrin (Merck MABS2155), while wax‐embedded sections were used for haematoxylin and eosin and Masson's Trichrome staining.

### 4.26 Explant Histology and Immunostaining

For immunostaining, frozen sections were thawed at room temperature and blocked with 0.3% (v/v) Triton X‐100 and 5 mg mL^−1^ BSA in PBS for 60 min. Sections were incubated overnight at 4°C with primary antibodies (1:100) diluted in PBS containing 5 mg mL^−1^ BSA and 0.1% (v/v) Triton X‐100. After washing in PBS and PBS‐T (PBS with 0.01% v/v Triton X‐100), slides were incubated with secondary antibodies (1:200, 2 h, room temperature), counterstained with DAPI (2 µg mL^−1^, 10 min, room temperature), and washed again in PBS‐T and PBS. Sections were mounted in ProLong Gold Antifade Mountant (Merck P10144) and coverslipped. For haematoxylin and eosin staining, paraffin sections were dewaxed in xylene (30 min) and rehydrated through graded industrial methylated spirits (100%, 90%, 70%) to water. Slides were stained with Ehrlich's haematoxylin (5 min), differentiated in acid alcohol, blued in Scott's tap water substitute, and rinsed in tap water between steps. Sections were counterstained with 1% aqueous eosin (10 s), washed, dehydrated, cleared in xylene, and mounted in DPX. For Masson's Trichrome staining, paraffin sections were dewaxed in Histoclear (30 min), rehydrated through graded alcohols to water, and then soaked overnight in Bouin's fixative. After thorough washing, slides were stained in Weigert's haematoxylin (5 min), differentiated in acid alcohol, blued in Scott's tap water substitute, and rinsed in tap water. Sections were then stained in Masson's red (∼1 min), rinsed in distilled water, and differentiated in phosphomolybdic acid until the collagen appeared colorless and muscle appeared pink. After rinsing in distilled water, slides were stained in Masson's blue for collagen visualization, washed rapidly in distilled water, air dried, cleared in xylene (5 min), and mounted in DPX. All slides were imaged with an Evident VS200 slide scanner with a 20X objective lens and DAPI, FITC, and Cy3 filters.

### 4.27 Statistical analysis

Statistical analysis and plotting of rheological experiments and compression tests were performed using Origin 2024b (OriginLab). Statistical analysis and plotting of biological experiments were performed using GraphPad Prism 10. Shapiro‐Wilk tests were performed to assess normality. Statistical significance was assessed using either a Kruskal‐Wallis test with Dunn's post‐hoc test, an unpaired t‐test with Welch's correction, Fisher's exact test, or a two‐way ANOVA with Tukey post‐hoc test, unless stated otherwise in the figure captions.

## Conflicts of Interest

The authors declare no conflict of interest.

## Supporting information




**Supporting File 1**: adhm70640‐sup‐0001‐SuppMat.docx.

**Supporting File 2**: adhm70640‐sup‐0002‐VideoS1.mp4.

**Supporting File 3**: adhm70640‐sup‐0003‐VideoS2.mp4.

**Supporting File 4**: adhm70640‐sup‐0004‐VideoS3.mp4.

## Data Availability

Raw data is available at data.bris.
