# Supplementary material for: Coagulative Granular Hydrogels with an Enzyme Catalyzed Fibrin Network for Endogenous Tissue Regeneration
Source: Adv Healthc Mater. 2025 Dec 21;15(27):e04146. doi: 10.1002/adhm.202404146 (PMC13378473; doi:10.1002/adhm.202404146)
Supplement: Supplementary file 1 — Supporting File 1: adhm70640‐sup‐0001‐SuppMat.docx. [file ADHM-15-0-s003.docx]

Supporting Information

Coagulative Granular Hydrogels with an Enzyme Catalyzed Fibrin Network for Endogenous Tissue Regeneration

Zhipeng Deng, Camila B. Tovani, Simona Bianco, Roxana Moscalu, Gianni Comandini, Aya Elghajiji, Mina Aleemardani, Jeremie Zappia, Bianca Fernandes, Olivia Annett, Chrissy L. Hammond, Jason Wong, Dave J. Adams, Fabrizio Scarpa, Michael R. Whitehouse, Annela M. Seddon, James P. K. Armstrong*


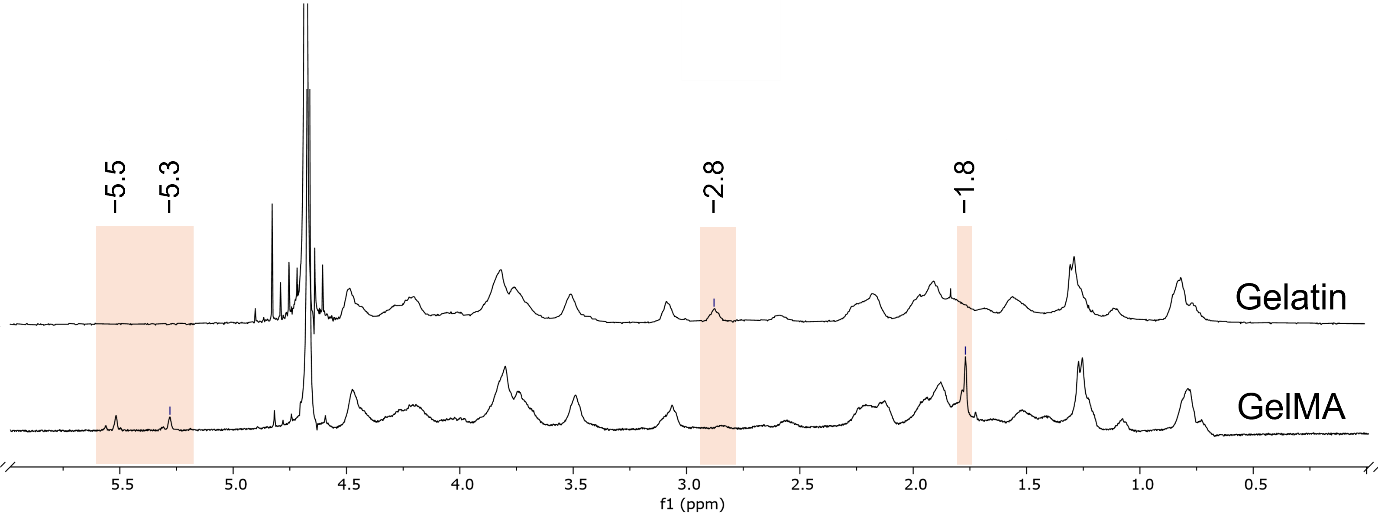


**Figure S1**. Representative ^1^H NMR spectra of GelMA and gelatin. The spectrum of GelMA displayed peaks corresponding to acrylic protons (2H) of methacrylamide on lysine sidechains (~5.3 ppm) and hydroxylysine sidechains (~5.5 ppm), and methyl protons (3H) of methacrylamide on either lysine or hydroxylysine sidechains (~1.8 ppm). These three peaks, which were not present in gelatin, indicated a successful conjugation of methacryloyl groups. The near absence a methylene lysine proton peak (2H) (~2.8 ppm), which was present in the spectrum of gelatin, indicated that the free amino groups of gelatin were efficiently reacted.^[1]^


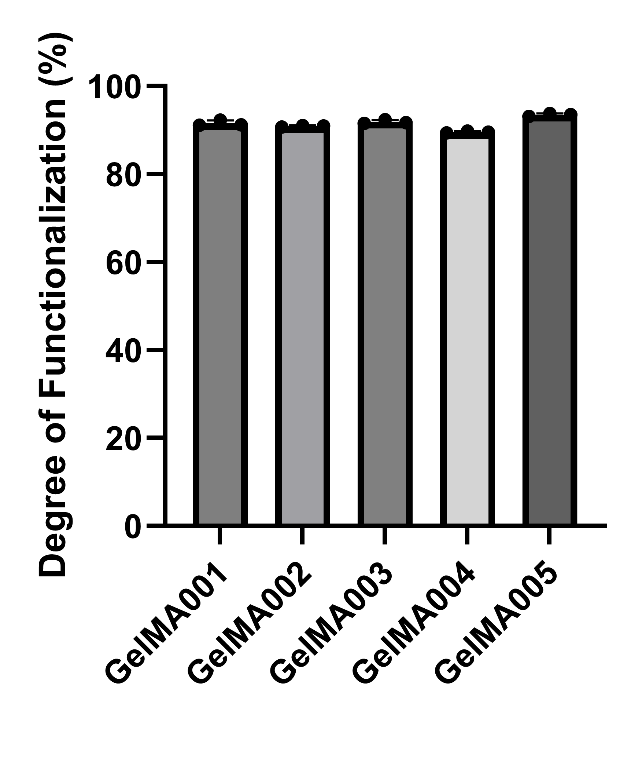


**Figure S2**. The degree of functionalization of the five GelMA batches (labelled 001-005) used in this study, which was calculated from a fluoraldehyde assay against gelatin standards. Data shown as mean ± standard deviation, n = 3.


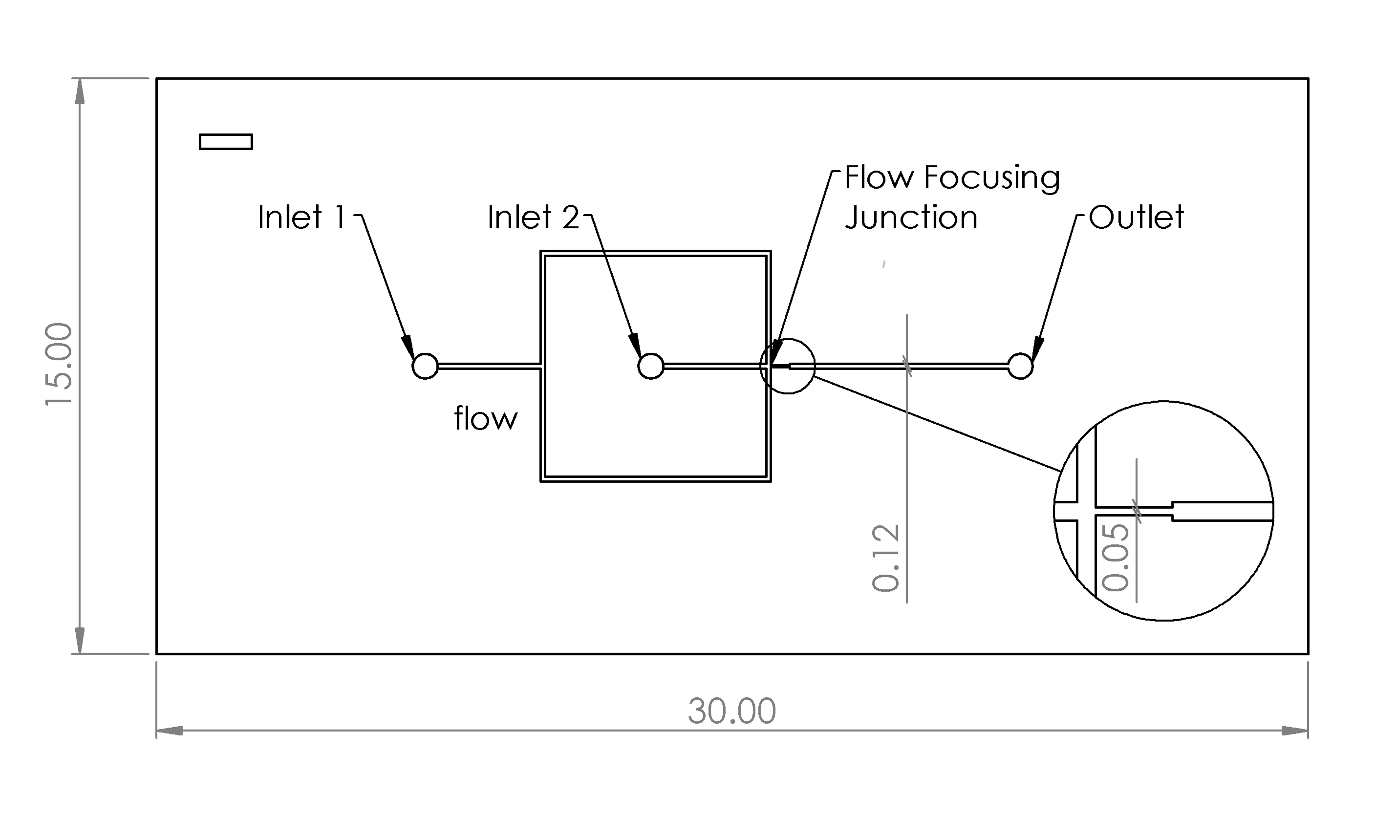


**Figure S3**. Design of the microfluidic chip for microgel fabrication. The surfactant-supplemented oil phase enters via inlet 1 and the liquid hydrogel precursor phase enters via inlet 2. The two liquids meet at the flow focusing junction, which generates hydrogel precursor microdroplets in a continuous flow of oil. The microgel droplets exit via the outlet and are then crosslinked with UV radiation. The channel has a constant width of 0.12 mm, except at the orifice of the flow focusing junction, where it narrows to 0.05 mm. Units in mm.


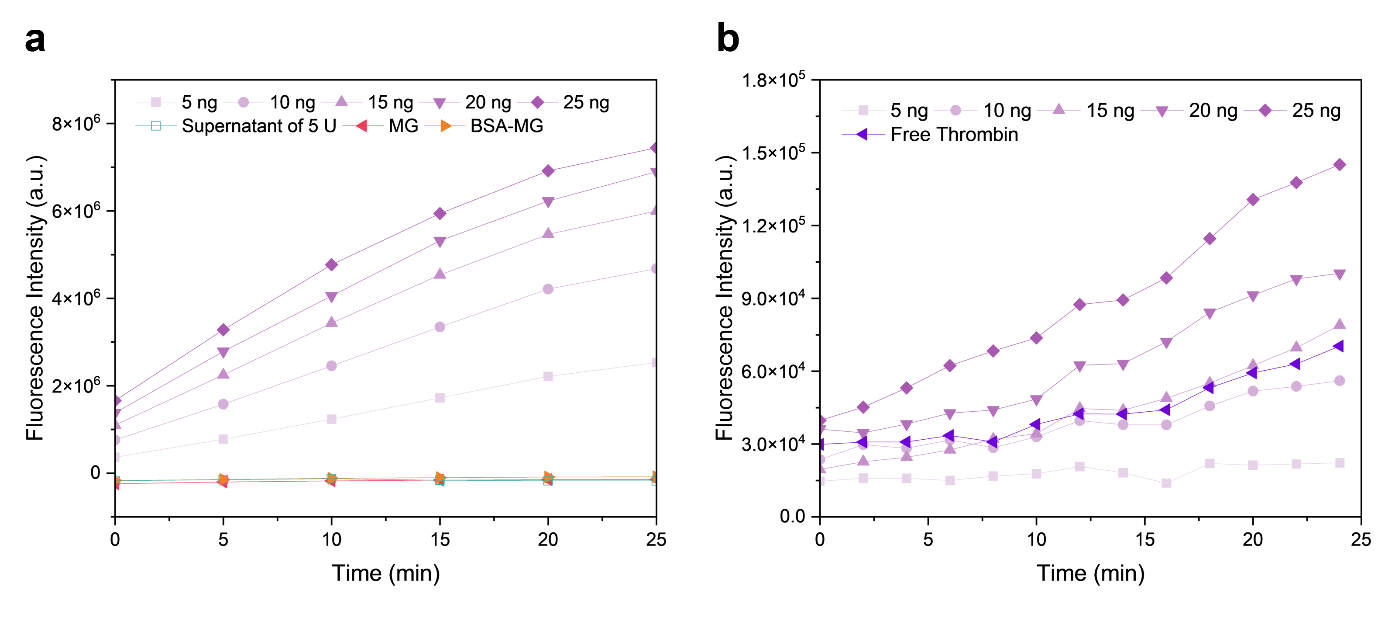


**Figure S4**. Thrombin activity assay. a) Activity assay curves of all controls: unfunctionalized microgels, BSA-functionalized microgels, and the supernatant from a 5 U thrombin functionalization reaction. When compared to the thrombin standards, these controls all showed negligible enzyme activity. b) Activity assay curve of thrombin reagent solution incubated overnight against fresh thrombin standards. The calculated activity is ~10 ng, which is 43% of the activity (23 ng) of fresh thrombin at the same concentration.


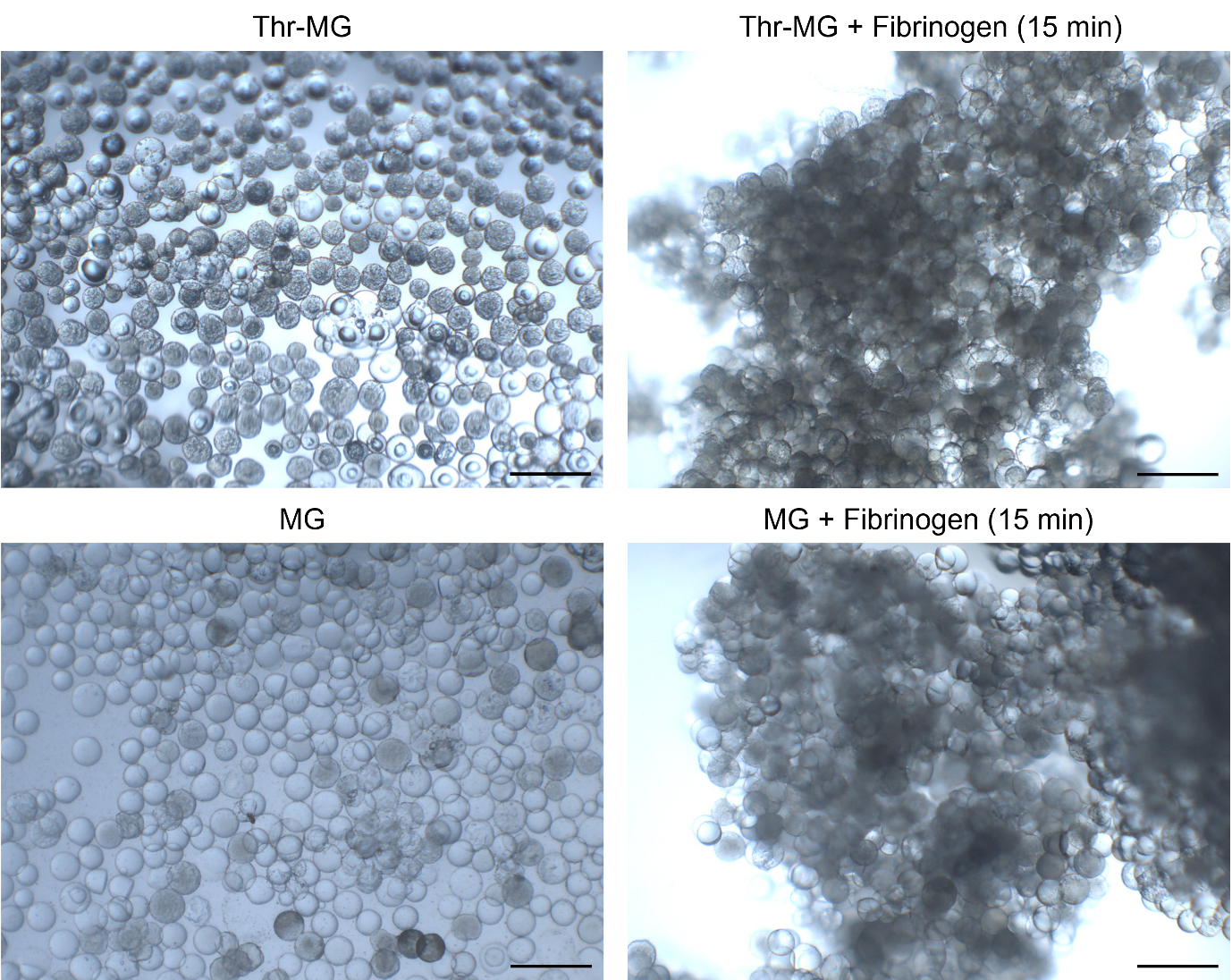


**Figure S5**. Optical images of Thr-MG and unfunctionalized microgels (MG) without any fibrinogen and 15 minutes after the addition of fibrinogen. Aggregation of microgels was observed in both groups at the early timepoint. Scale bar: 200 µm.


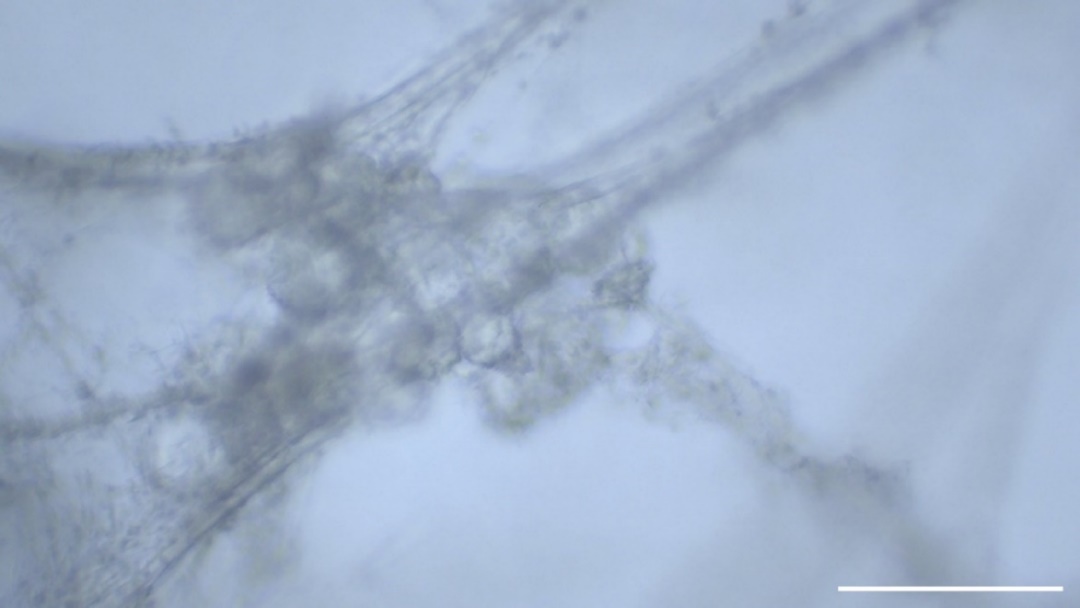


**Figure S6**. Brightfield image of Thr-MG aggregates incubated with fibrinogen for 24 h showed the presence of fibers, assumed to be fibrin, entangled with the Thr-MGs. Scale bar: 200 µm.


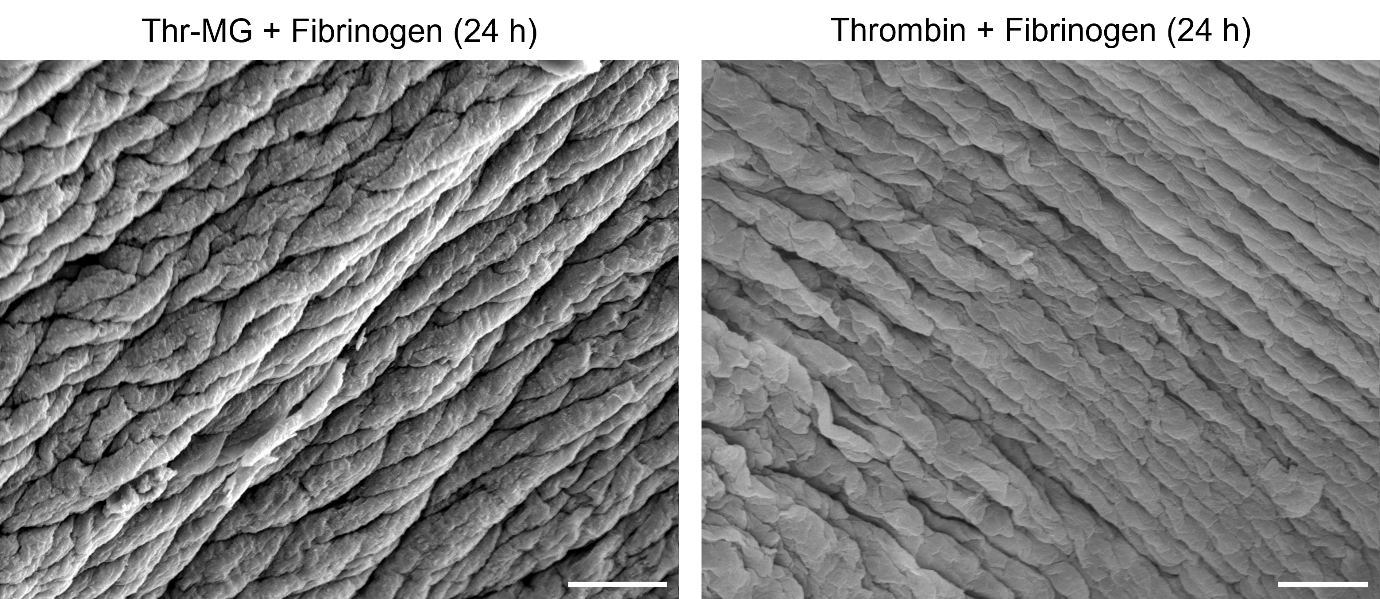


**Figure S7**. Representative scanning electron microscopy images of fibrin formed by Thr-MG (left) and thrombin solution (right). These samples showed similar morphology, with both exhibiting fibrillar structure and hierarchical organization. Scale bar: 10 µm.


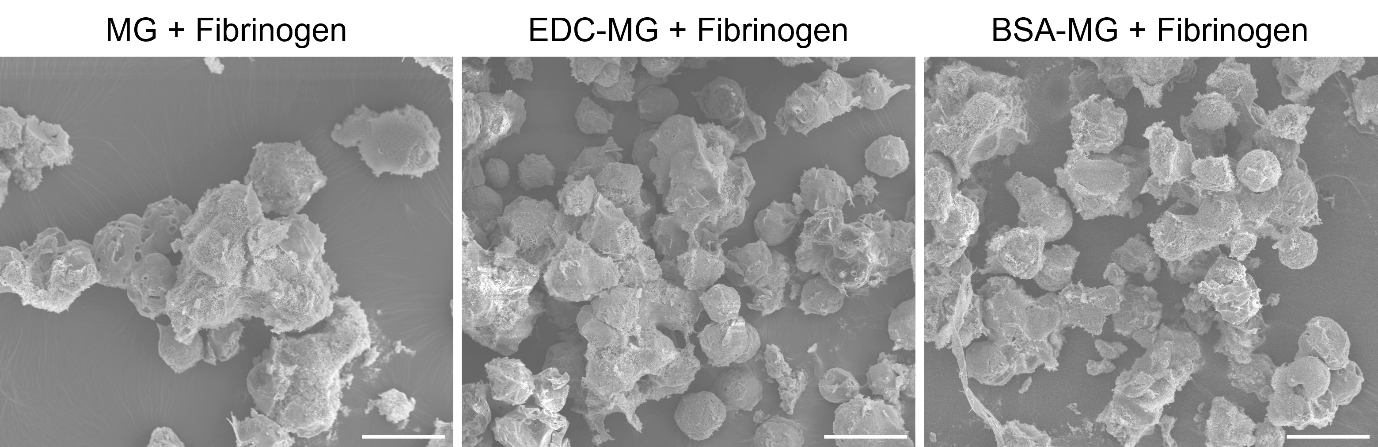


**Figure S8**. Representative scanning electron microscopy images of microgels (MG), EDC-activated microgels (EDC-MG), BSA-functionalized microgels (BSA-MG), each incubated with fibrinogen for 24 h. Scale bar: 100 µm.


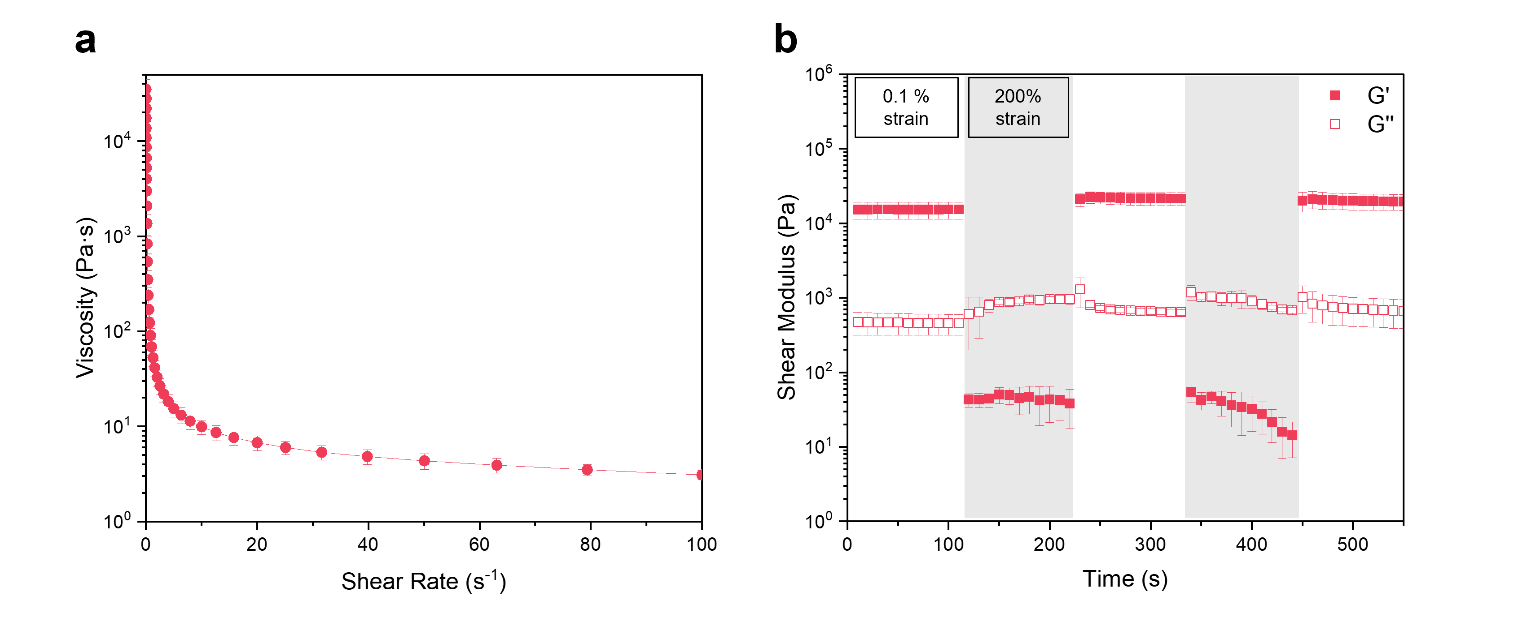


**Figure S9**. a) Viscosity measurement of unfunctionalized granular hydrogels (GH) with shear rates from 0-100 s^-1^. b) Shear recovery test of GH, measuring shear storage modulus (G') and shear loss modulus (G'') when alternating between 0.1% strain (unshaded) and 200% strain (shaded). Data shown as mean ± standard deviation, n = 3.


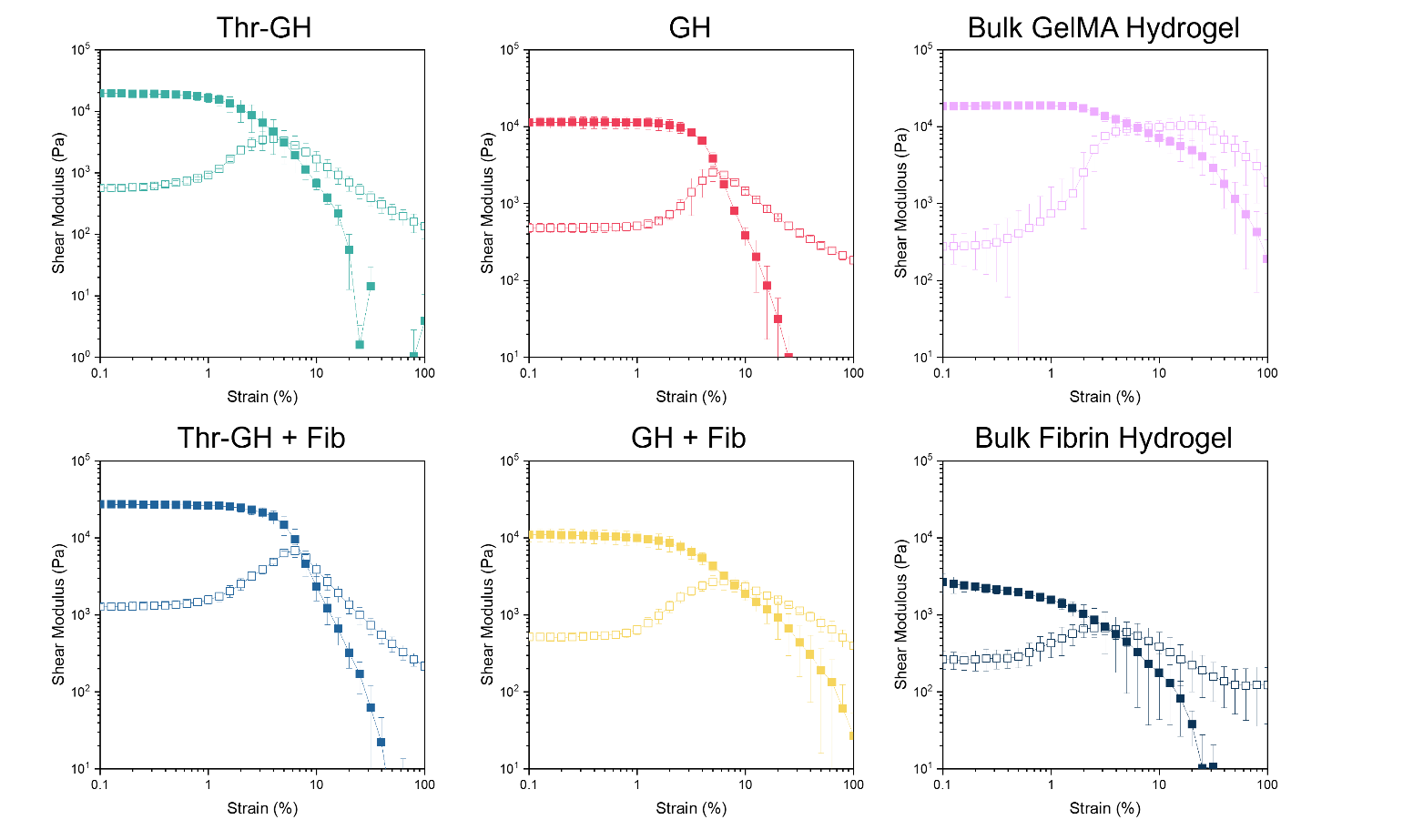


**Figure S10**. Oscillatory shear strain sweeps of Thr-GH, Thr-GH-fibrin composite (Thr-GH + Fib), GH, GH with the addition of fibrinogen (GH + Fib), bulk GelMA hydrogel, and bulk fibrin hydrogel. The shear storage modulus (G', filled symbols) and shear loss modulus (G'', open symbols) were measured across a shear strain range of 0.1-100%. Data shown as mean ± standard deviation, n = 3.


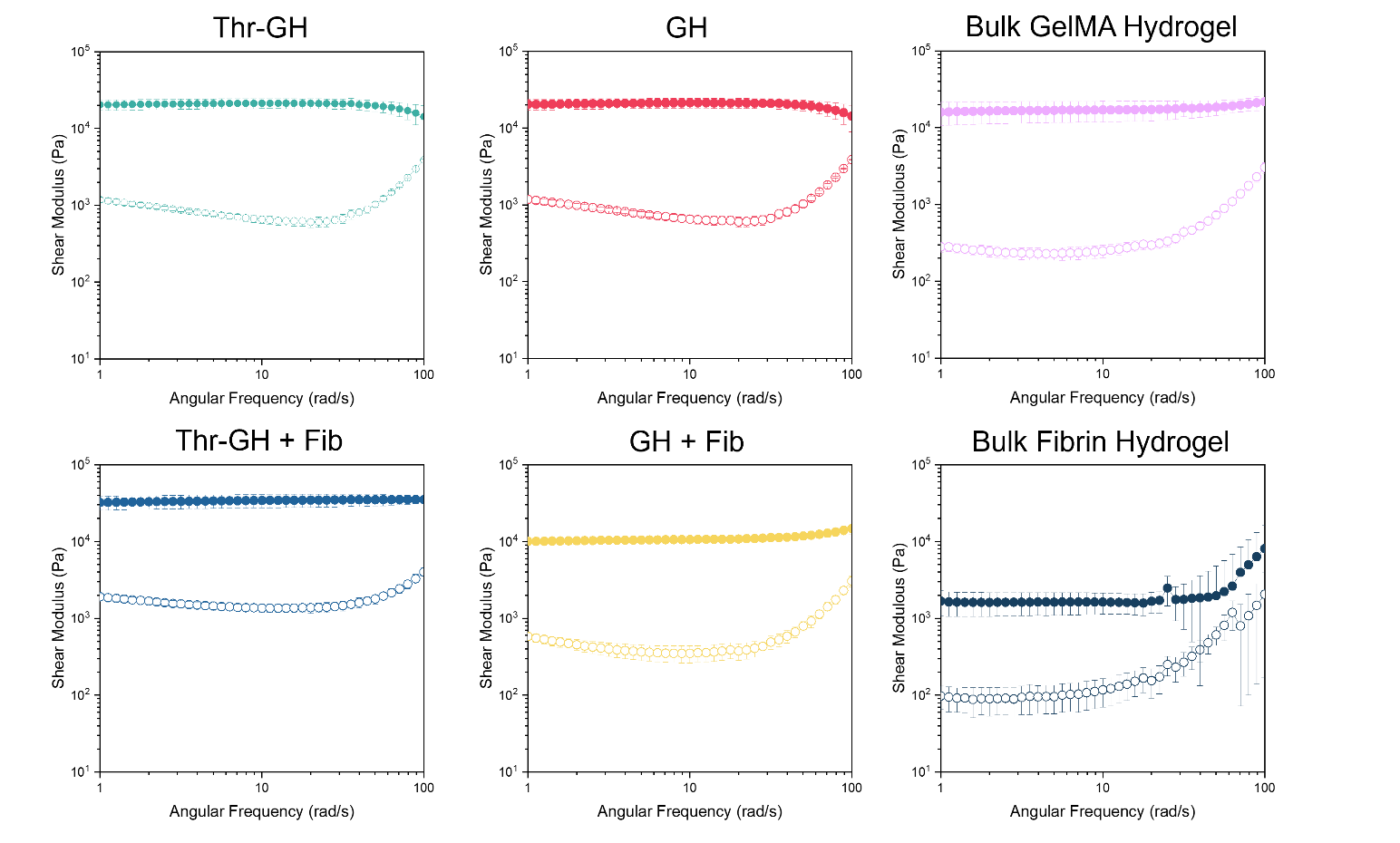


**Figure S11**. Oscillatory shear frequency sweeps of Thr-GH, Thr-GH + Fib, GH, GH + Fib, bulk GelMA hydrogel, and bulk fibrin hydrogel. The shear storage modulus (G', filled symbols) and shear loss modulus (G'', open symbols) were measured across an angular frequency range of 1-100 rad/s. Data shown as mean ± standard deviation, n = 3.


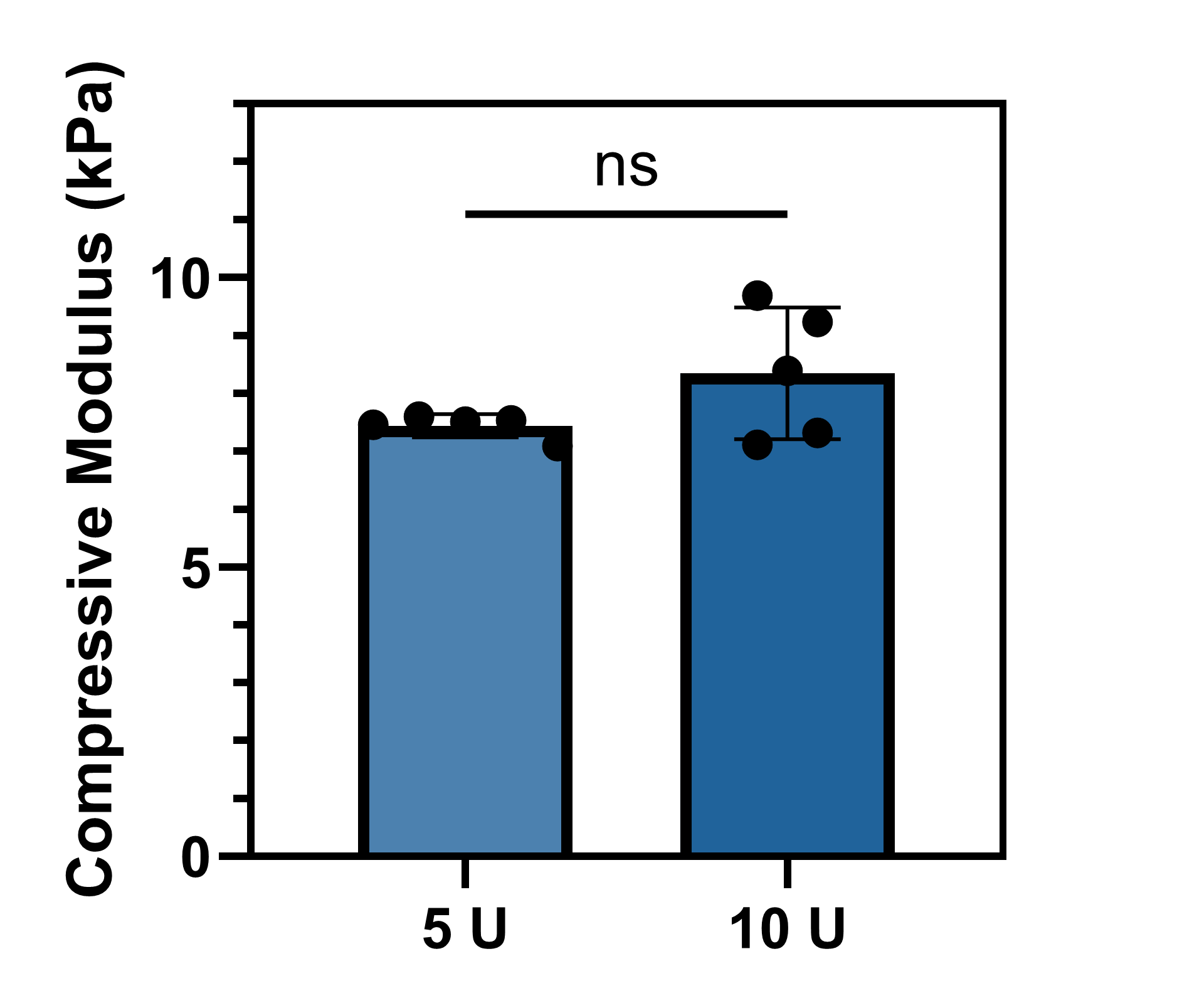


**Figure S12**. Comparison of the compressive modulus between two batches of Thr-GH + Fib samples functionalized with 5 U and 10 U of thrombin, respectively. Statistical analysis performed using Welch’s t-test. Data are presented as mean ± standard deviation, with a sample size of n = 5.

**
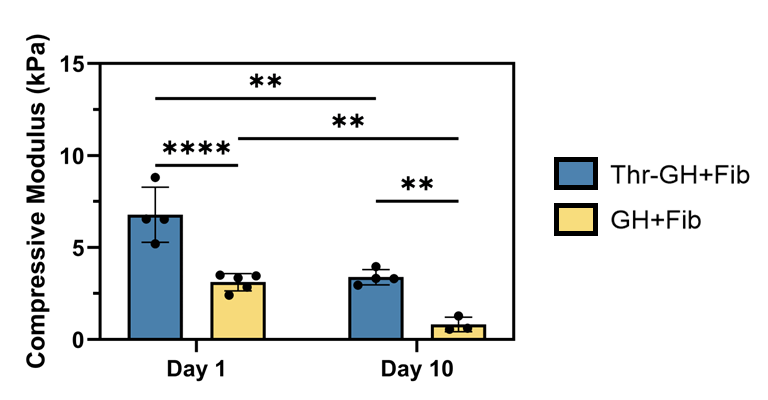
**

**Figure S13.** Changes in the compressive modulus of Thr-GH + Fib and GH + Fib samples before and after 10 days of incubation at 37℃, with moduli calculated as the slope between 2-7% strain. Two of the GH + Fib control samples were dissociated on day 10, so only three samples remained for testing. Statistical analysis performed using Two-Way ANOVA (mixed-effects model) with uncorrected Fisher’s LSD. ****p < 0.0001, **p < 0.01, *p < 0.05. Data are presented as mean ± standard deviation, with a sample size of n ≥ 3.


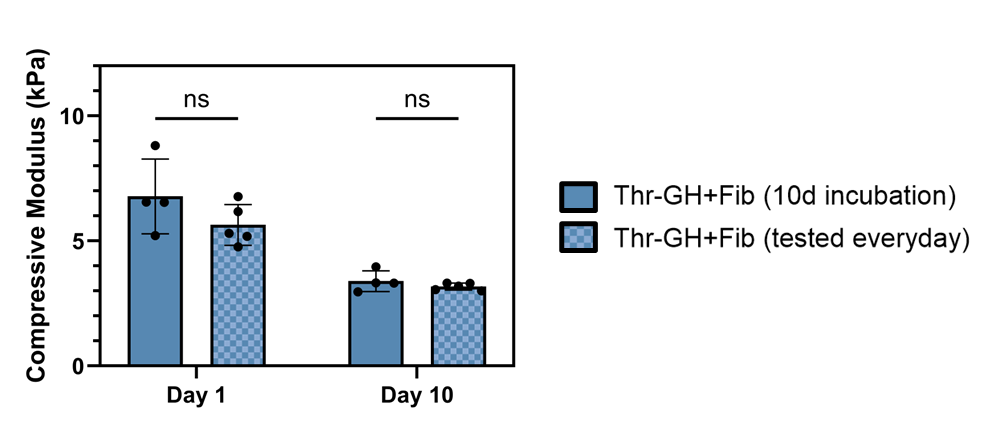


**Figure S14**. Comparison of the compressive modulus of Thr-GH + Fib and GH + Fib samples, prepared fresh and after 10 days of passive degradation (incubation at 37℃) or daily dynamic loading (compression applied once per day for 10 days). Statistical analysis performed using Two-Way ANOVA (mixed-effects model) with Šidák’s post-hoc test. Data are presented as mean ± standard deviation, with a sample size of n ≥ 4.


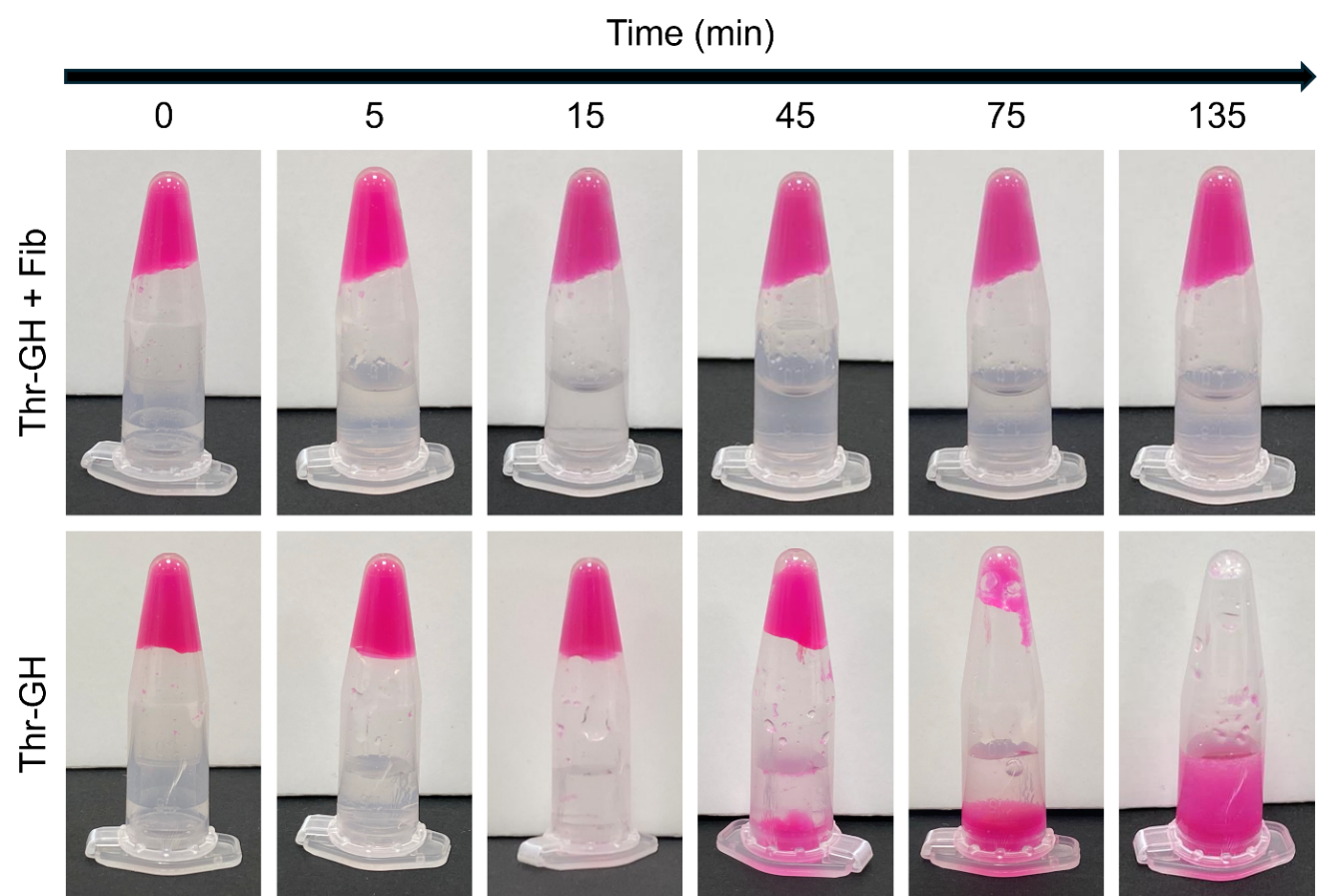


**Figure S15**. The stability of Thr-GH + Fib and Thr-GH control was assessed by exposing the samples to continuous agitation. The Thr-GH + Fib composite remained intact under the dynamic conditions, while the Thr-GH control began to disintegrate after 15 min of agitation and was completely dissociated after 2 h.


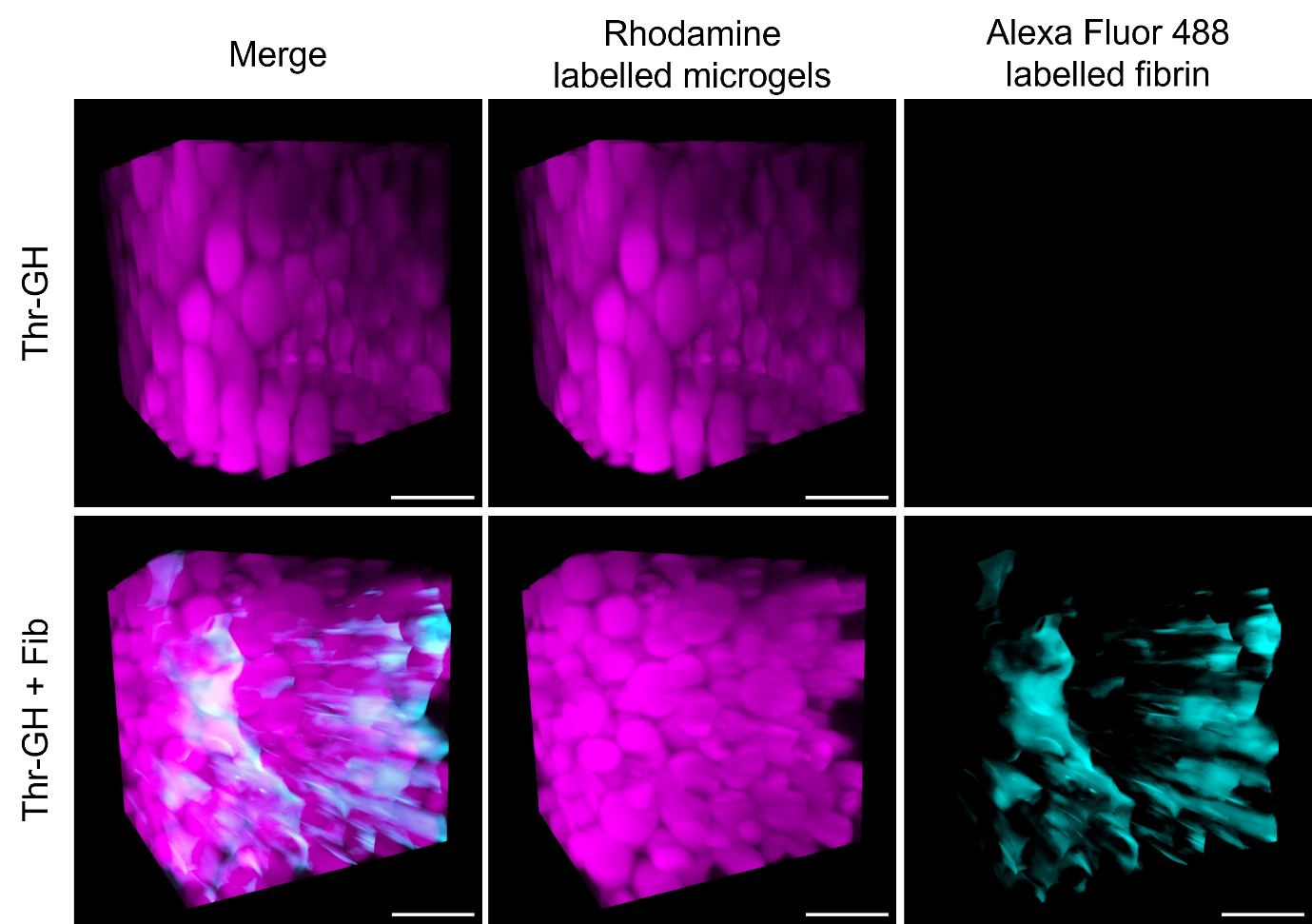


**Figure S16**. 3D reconstructed stacks of Thr-GH (top panel) and Thr-GH + Fib (bottom panel) from light sheet fluorescence microscopy. Microgels were made with GelMA fluorescently labelled with rhodamine (shown in purple), while the Thr-GH sample was incubated with Alexa Fluor 488 labelled fibrinogen (shown in cyan). Scale bar: 500 µm.


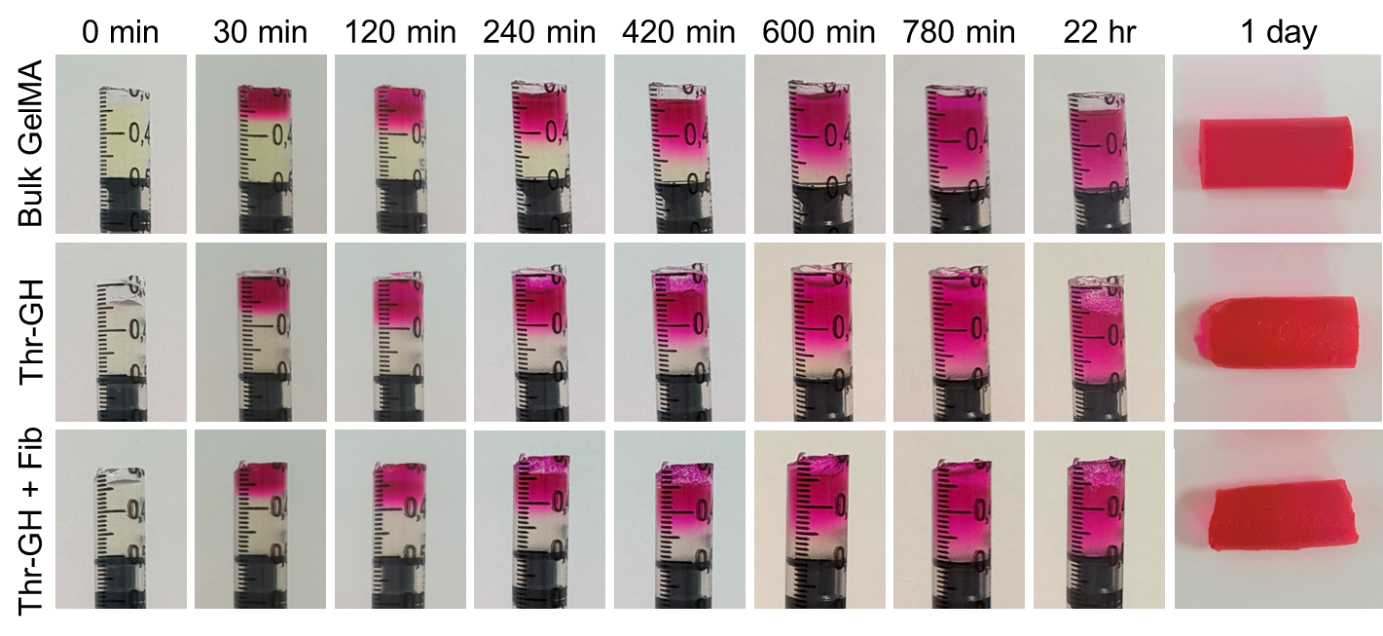


**Figure S17**. Camera images showing infiltration of Rhodamine B (0.5 kDa) into Thr-GH + Fib, compared to bulk GelMA and Thr-GH controls.


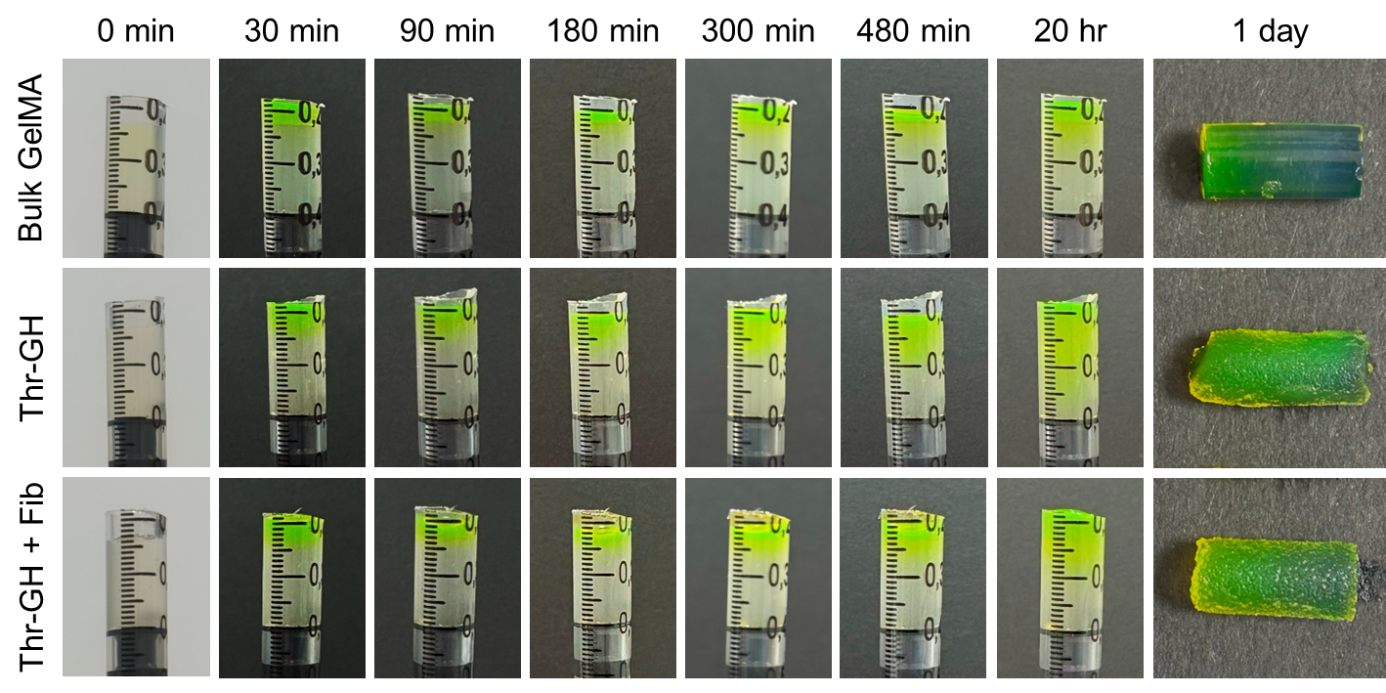


**Figure S18**. Camera images showing infiltration of FITC-labeled dextran (40 kDa) into Thr-GH + Fib compared to bulk GelMA and Thr-GH controls.


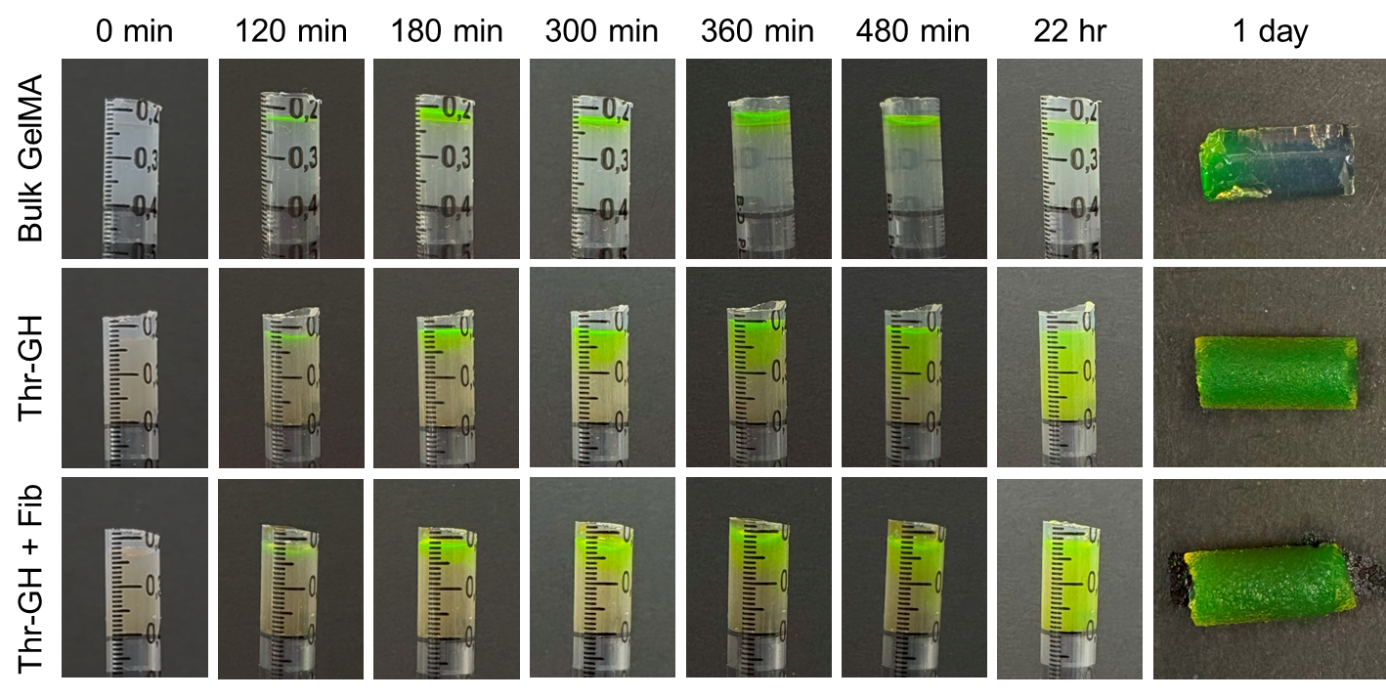


**Figure S19**. Camera images showing infiltration of FITC-labeled dextran (150 kDa) into Thr-GH + Fib, compared to bulk GelMA and Thr-GH controls.


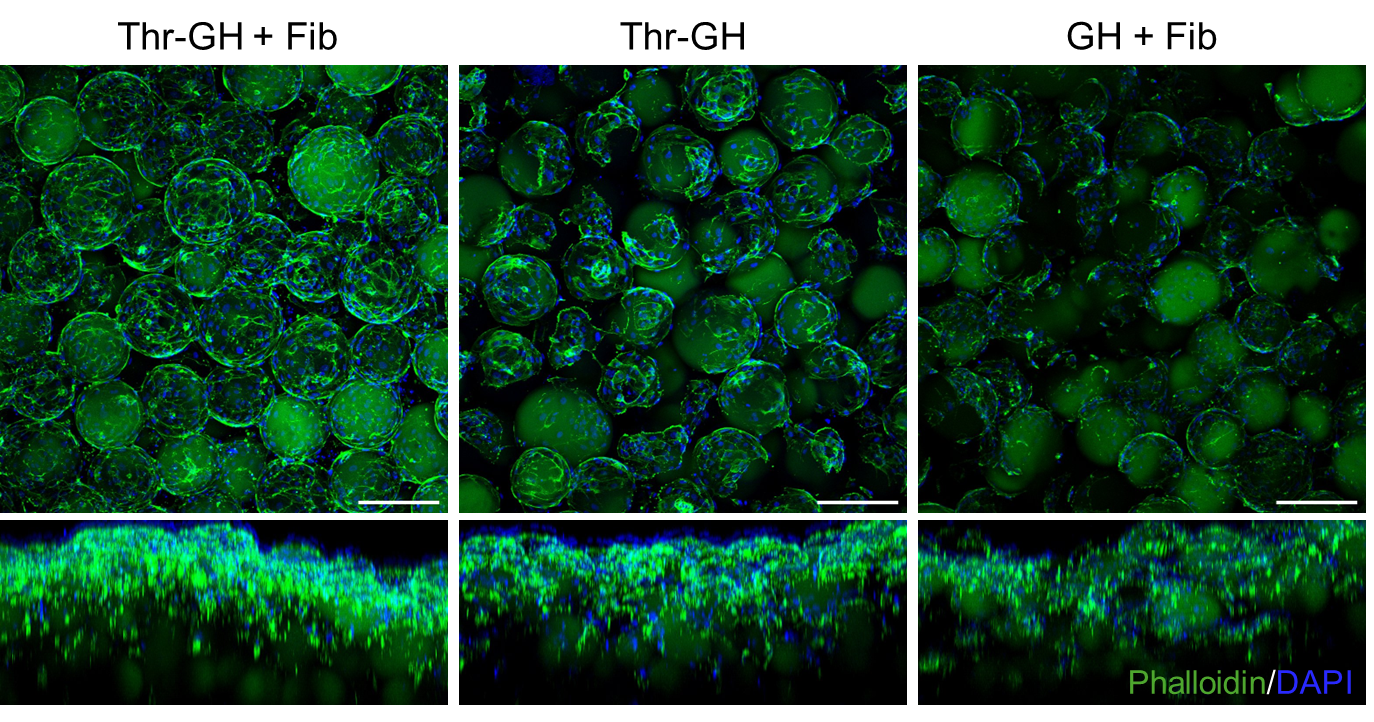


**Figure S20**. Representative maximum intensity projection images of HUVECs cultured for 1 day on Thr-GH + Fib, compared to Thr-GH and GH + Fib controls. Images were resliced to visualize cell infiltration and distribution within the samples and controls. Scale bar: 200 µm.


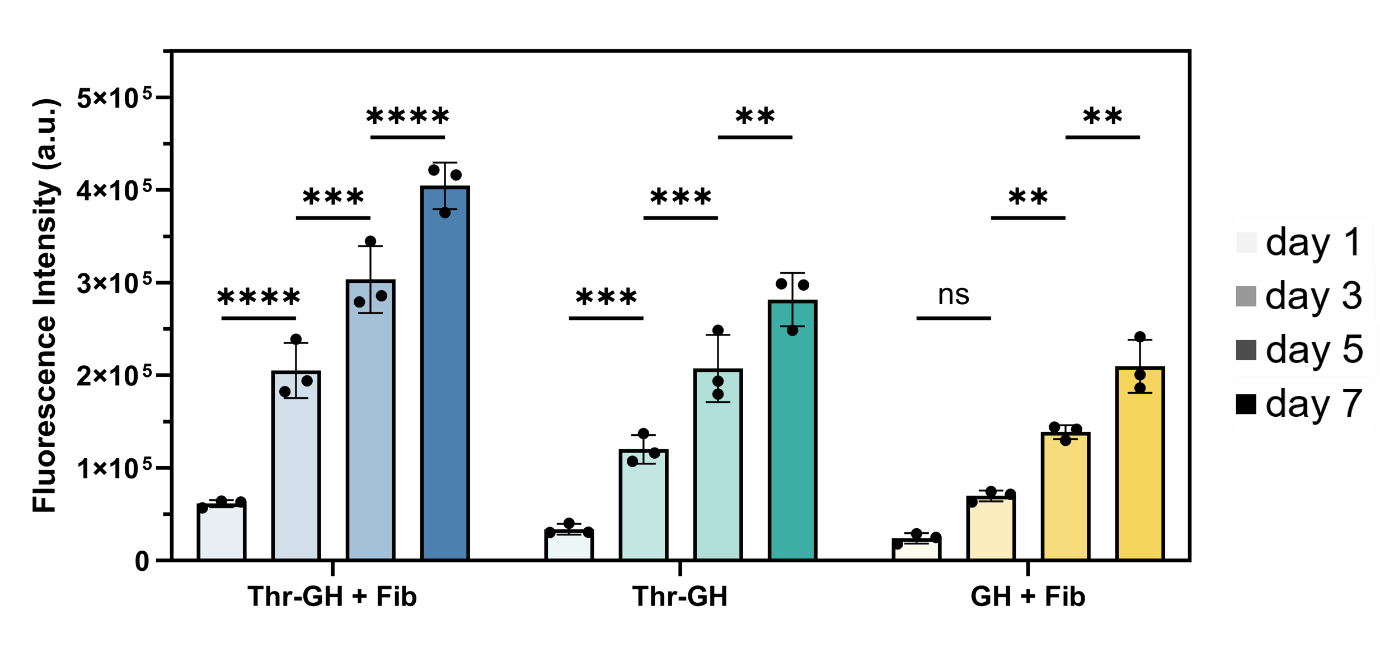


**Figure S21**. Quantification of cell metabolism in Thr-GH + Fib, Thr-GH and GH + Fib over 7 days. Data are presented as mean ± standard deviation, with a sample size of n = 3. Statistical analysis performed using two-way ANOVA with Tukey’s post-hoc test; ns = no significance (p > 0.05), **p < 0.01, ***p < 0.001, ****p < 0.0001.


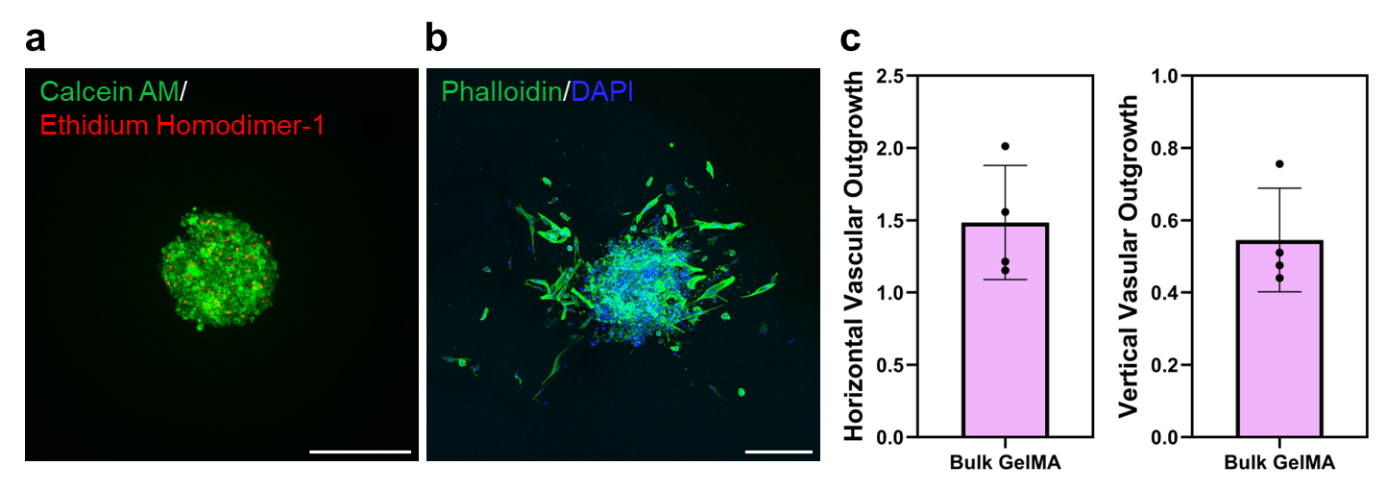


**Figure S22**. a) Representative fluorescence microscopy image of a LIVE/DEAD-stained vascular spheroid before seeding into the granular hydrogel (calcein AM for viable cells, green; ethidium homodimer-1 for non-viable cells, red). Scale bar: 200 µm. b) Representative maximum projection image of a spheroid cultured in a bulk GelMA hydrogel cavity for 3 days. The spheroid was stained for F-actin filaments (phalloidin, green) and nuclei (DAPI, blue). Scale bar: 200 µm. c) Quantification of the horizontal and vertical outgrowth in bulk GelMA hydrogels, with the average radii of the day 3 vascular networks normalized to a day 0 spheroid. Data are presented as mean ± standard deviation, with a sample size of n = 4.


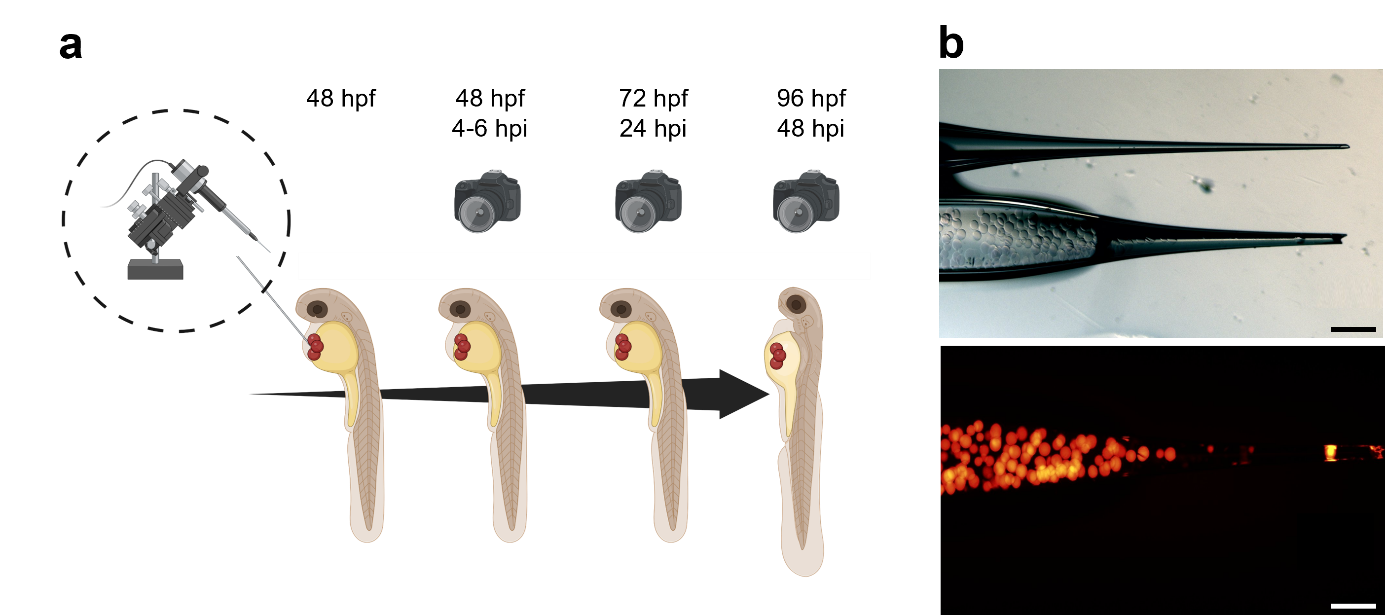


**Figure S23.** Experiment design and setup of the zebrafish study. a) Schematic representation of the study design. Larvae were injected at 48 hours post fertilisation (hpf) and live imaging was performed at 4-6, 24, and 48 hours post injection (hpi). b) Representation of a needle for typical microinjection in zebrafish (top) and a needle used for the injection of the granular hydrogel (bottom), with an opening of >100 µm. Scale bar: 500 µm.

**SUPPLEMENTARY REFERENCES**

[1] H. Shirahama, B. H. Lee, L. P. Tan, N. Cho, *Sci. Rep.* **2016**, *6*, 31036.
